# Eggshell waste bioprocessing for sustainable acid phosphatase production and minimizing environmental hazards

**DOI:** 10.1186/s13036-024-00421-8

**Published:** 2024-04-08

**Authors:** Soad Abubakr Abdelgalil, Mohamed Mohamed Yousri Kaddah, Gaber Attia Abo-Zaid

**Affiliations:** 1 Bioprocess Development Department, Genetic Engineering and Biotechnology Research Institute (GEBRI), City for Scientific Research and Technological Applications (SRTA‑City), Universities and Research Institutes Zone, Alexandria, New Borg El‑Arab City 21934 Egypt; 2 Pharmaceutical and Fermentation Industries Development Center, City for Scientific Research and Technological Applications (SRTA‑City), Universities and Research Institutes Zone, Alexandria, New Borg El‑Arab City 21934 Egypt

**Keywords:** Chicken eggshell waste, Acid phosphatase, *Bacillus sonorensis*, Bioremediation, Statistical experimental design, Batch fermentation, Organic acids, Bioreactor

## Abstract

**Background:**

The Environmental Protection Agency has listed eggshell waste as the 15th most significant food industry pollution hazard. Using eggshell waste as a renewable energy source has been a hot topic recently. Therefore, finding a sustainable solution for the recycling and valorization of eggshell waste by investigating its potential to produce acid phosphatase (ACP) and organic acids by the newly-discovered *B. sonorensis* was the target of the current investigation.

**Results:**

Drawing on both molecular and morphological characterizations, the most potent ACP-producing *B. sonorensis* strain ACP2, was identified as a local bacterial strain obtained from the effluent of the paper and pulp industries. The use of consecutive statistical experimental approaches of Plackett–Burman Design (PBD) and Orthogonal Central Composite Design (OCCD), followed by pH-uncontrolled cultivation conditions in a 7 L bench-top bioreactor, revealed an innovative medium formulation that substantially improved ACP production, reaching 216 U L^−1^ with an ACP yield coefficient *Y*_*p/x*_ of 18.2 and a specific growth rate (*µ*) of 0.1 h^−1^. The metals Ag^+^, Sn^+^, and Cr^+^ were the most efficiently released from eggshells during the solubilization process by *B. sonorensis.* The uncontrolled pH culture condition is the most suitable and favoured setting for improving ACP and organic acids production. Quantitative and qualitative analyses of the produced organic acids were carried out using liquid chromatography-tandem mass spectrometry (LC–MS/MS). Lactic acid, citric acid, and hydroxybenzoic acid isomer were the most common organic acids produced throughout the cultivation process. The findings of TGA, DSC, SEM, EDS, FTIR, and XRD analysis emphasize the significant influence of organic acids and ACP activity on the solubilization of eggshell particles.

**Conclusions:**

This study emphasized robust microbial engineering approaches for the large-scale production of a newly discovered acid phosphatase, accompanied by organic acids production from *B. sonorensis*. The biovalorization of the eggshell waste and the production of cost-effective ACP and organic acids were integrated into the current study, and this was done through the implementation of a unique and innovative medium formulation design for eggshell waste management, as well as scaling up ACP production on a bench-top scale.

**Supplementary Information:**

The online version contains supplementary material available at 10.1186/s13036-024-00421-8.

## Background

There has been a warning signal of the industrial revolution, and that is an increase in waste production, which has brought toxic and hazardous wastes to the attention of the scientific community [1]. Agro-food from industrial and community fields contributes substantially to pollution, necessitating the address of this problem. As environmental regulations and laws become more stringent, it is essential to seek out and develop innovative methods for the management of agro-food wastes. Disposing of the massive volumes of trash produced by the food processing industry is challenging. Converting trash into valuable commodities for sustainable development is critical in this era of ever-increasing efforts to turn waste into wealth. Wastes should be recycled, reused, and directed towards producing valuable and utilizable products to achieve sustainable development.

On the one hand, this is to safeguard the environment; on the other, it is to acquire value-added products while earning a zero-waste standard. Waste utilization is a top priority nowadays for sustainable development [2]. Eggshell waste is considered dangerous by the European Commission and ranked as the fifteenth most polluting food processing issue by the Environmental Protection Agency [3]. Eggshells account for approximately 11 percent of the total weight of the egg. Their composition consists of a network of protein fibers linked by calcium carbonate crystals (which accounts for more than 96 percent of the shell weight), magnesium carbonate (1 percent), calcium phosphate (1 percent), magnesium phosphate (1 percent), silicon oxide (1 percent), and other trace amounts of different elements and organic compounds. According to the context of the circular economy, recycling or valorizing this waste, rather than disposing of it in landfills, offers an opportunity for environmental protection and preservation of natural resources [4].

**Fig. 1:**
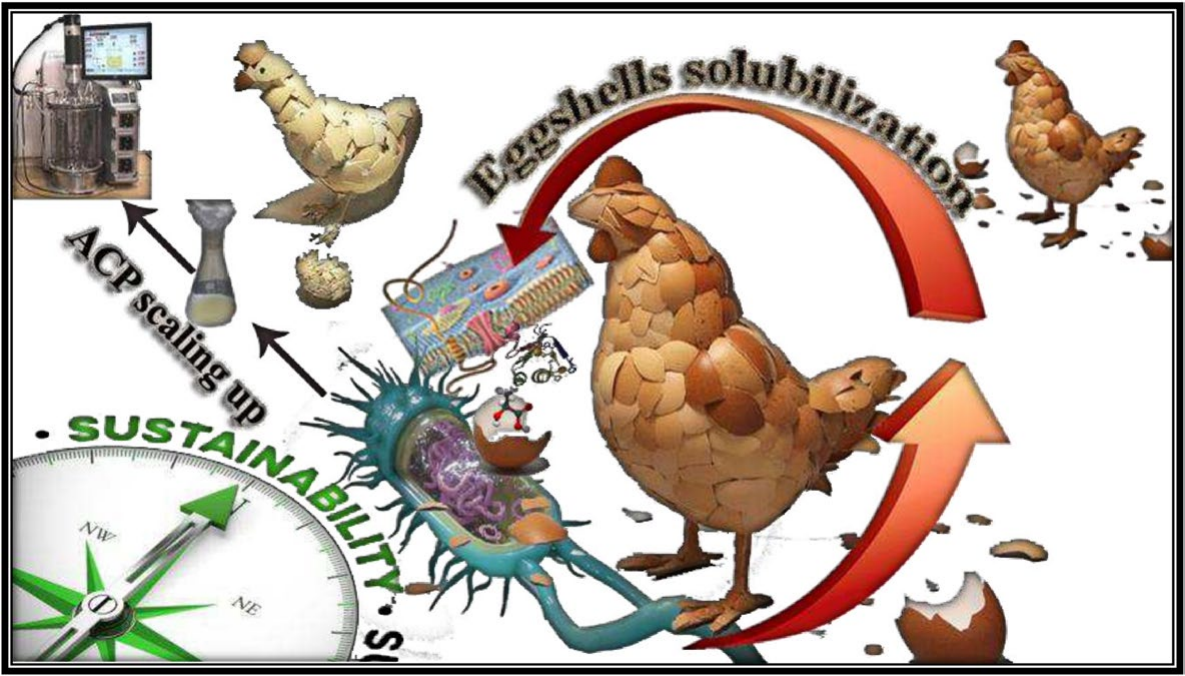
A schematic illustration demonstrating the ultimate benefit of exploiting distinctive features of the newly discovered *B. sonorensis* strain ACP2 for bio-solubilization of eggshell waste and boosting ACP production as green sustainability approaches at a bench-top bioreactor production scale

Chicken eggshells have been the subject of many studies regarding bioresource recovery and reuse in biological, chemical, engineering, and environmental technologies for producing value-added products. Despite this, as the authors know, no research has been done on eggshell waste as a nutritive source of microbial growth for producing industrial microbial enzymes. The need for industrial enzymes is increasing continually, driven by a rising desire for eco-friendly alternatives [5]. Incentivizing the hydrolysis of phosphor-monoesters at an acidic pH are acid phosphatases (Orthophosphoric monoester phosphohydrolase, EC 3.1.3.2), a family of enzymes that are widely distributed. These enzymes are present in every bacteria and are responsible for the breakdown of various phosphomonoesters and the transphosphorylation reactions, which include the transfer of some or all phosphoryl units to alcohol with the help of certain phosphate recipients [6]. Activated phospho-enzyme intermediates are synthesized during active catalysis and may share their phosphate group with water, glucose, or inosine. Bacterial acid phosphatase is used to synthesise hydroxyl apatite, mineralization of organic phosphorous, waste cleanup, metal recovery, antagonistic action against plant diseases, and plant growth stimulation [7, 8]. It takes constant effort to enhance industrial enzyme production under ideal circumstances to produce enzymes with the required properties. A fermentation process's capacity for production is mainly determined by the strains employed in it, which are influenced by interactions between these strains' physiological responses and the scale-dependent flow fields produced inside bioreactors. Understanding and deciphering these intricate interactions is essential for a better understanding the fermentation process as it scales up [9]. Therefore, no findings have been published about the biovalorization of waste chicken eggshells utilizing a bioprocess innovation framework to increase acid phosphatase production. By employing the recently identified *Bacillus sonorensis* strain ACP2, the current contribution seeks to clarify the performance evaluation of an innovative conceptual bioprocess for ACP synthesis, and this report is the first to take an active role in combining ACP production with eggshell biovalorization using an innovative medium formulation design. Also, as far as the authors are aware, it was a crucial breakthrough in expanding ACP production from shake-flask to bench-top bioreactor scales, as illustrated in Fig. 1.

## Material and methods

### Sample collection and isolate sources

Handy•Alex Converta, for paper and pulp industries in Alexandria, Egypt, is the target factory for collecting wastewater samples aseptically. Upon arrival at the Bioprocess Development laboratory, the samples were moved to a refrigerator, where they were maintained at 4.0°C until they were utilized in the subsequent analysis.

### ACP-producing bacterial enrichment and isolation

Eggshell powder was added to Pikovskaya (PKV) broth medium instead of Ca_3_(PO_4_)_2_, pH 7.0, throughout the 72-h incubation period at 45 °C and 200 rpm to enrich the ACP-producing bacteria, as described by Abdelgalil et al. [10]. A bakery provided the eggshells, which were then repeatedly cleaned with distilled water to remove impurities. They were then blended and put through a 0.125-mm sieve after being dried for 3 h at 70 °C in an oven. Abdelgalil et al. [11] demonstrated that the enriched bacterial isolates were isolated, purified, and maintained on Luria–Bertani (LB) medium agar.

### Qualitative screening for ACP activity

To examine the isolates under investigation's capacity for producing acid and for solubilizing CaCO_3_, PKV's agar was modified with the mineral.; meanwhile, chromogenic substrate (tetrasodium salt of phenolphthalein diphosphate (PDP)) incorporated in LB broth media, were utilized as screening medium to assess ACP capability [12]. For screening the pure isolates, the activated pre-cultures were used to inoculate media agar plates and media broth of screening media, which were then incubated at 45 °C overnight. Among the intriguing isolates, ACP2 was chosen for further investigation and submitted to morphological and molecular identification procedures. This isolate was identified as having the highest intensity of purple hue and the largest solubilization clear zone.

### Quantitative screening for ACP activity

The quantitative evaluation of ACP activity was carried out as described by Abdelgalil et al. [13] The quantity of enzyme that generates 1.0 μmol *ρ*-nitrophenol in 1 min at pH 4.0 and 65 °C is given as an international unit (IU) and the activities were measured in U L^−1^ min^−1^.

### Amplification of the 16S rDNA gene, sequencing, and similarity

The most potent ACP-producing isolate's genomic DNA has been extracted utilizing the salting-out method [14]. The 16S rRNA gene segments were then amplified and sequenced, and a neighbor-joining phylogenetic tree diagram was developed for the evolutionary relationships [11].

### Morphological investigation of *B. sonorensis* strain ACP2

The City of Scientific Research and Technological Applications (SRTA-City) laboratory facilities' scanning electron microscopy (SEM) equipment was used to determine the top-performing ACP-producing bacteria's cell surface morphology and shape. A sputtering device (JFC-1100 E Joel, USA) was used to sputter a thin coating of gold over the dried bacterial thin film.

### Optimization of the physical parameters for the production of ACP

Using the quasi-optimum approach described by Abdelgalil et al., the effectiveness of acid phosphatase synthesis was studied for a range of physical parameters, such as temperature, beginning pH levels, and inoculum volume [15]. An investigation into the effects of temperature on the synthesis of ACP was undertaken by inoculating an isolation broth medium with an activated pre-culture derived from the most effective strain. The incubation period lasted 48 h, and the temperature was varied between 37, 40, and 45 °C while a rotary shaker operating at 200 rpm was utilized. The various activated inoculum quantities (1, 2, 3, 5, 10 percent) of the chosen strain were used as pre-inoculums of the aerobic culture of isolation broth medium at 45 °C and 200 rpm to figure out the most precise inoculum volume in the ACP production process.

The optimum pH for ACP synthesis was found using the most efficient temperature and inoculum size for ACP biosynthesis. Using 0.1 M HCl, the pH of 50 mL isolation broth medium was adjusted individually to 3.0, 4.0, 5.0, 6.0, 7.0, and 8.0. The isolation broth medium was also evaluated without the initial pH value adjustment. In a rotating shaker incubator, the inoculated flasks were incubated aerobically for 48 h at 45 °C (200 rpm). ACP activity was determined using the cell-free supernatants from each experiment.

### *B. sonorensis* strain ACP2 media composition for ACP biosynthesis

No standardized cultivation medium from various microbial sources was established for optimal ACP production. For that, in this research, statistical experimental design exploitations developed a novel approach to building media for ACP output.

### PBD

The significant nutrient parameters influencing ACP production and *B. sonorensis* (isolate ACP2) growth were determined using fractional factorial two-level models based on a 2^ k^-PBD (variables, k = 11) with six central points in 18 combination trial batches (twelve main batches plus six central point batches). To find out the most crucial factors influencing the maximum amount of ACP developed by the identified *B. sonorensis* in fermentation that was submerged, PBD chose at random the following materials: glucose, sodium glutamate, ammonium nitrate (NH_4_NO_3_), ammonium chloride (NH_4_Cl), eggshell powder, potassium chloride (KCl), magnesium sulfate (MgSO_4_•7H_2_O), and sodium phosphate (NaH_2_PO_4_), as well as the ammonium molybdate, chromium chloride (CrCl_3_•6H_2_O), and ferrous sulfate (FeSO_4_•7H_2_O) along with the activity of ACP selected as response with a level of assurance of all ranges at 95% to create regression coefficient estimates. There were two levels of investigation for each independent variable: high (+ 1) and low (-1) to evaluate the effect of each independent variable. The upper and lower boundaries of each variable's range were determined using these levels, and the six center points were used in six distinct batches for all of the independent variables Table 1. Statistical analysis was carried out on the acquired experimental data to ascertain the efficacy and efficiency of the regression model using statistical design available as free software. The first-order polynomial algorithm described below was used for statistical screening modelling [16].

**Table 1:** Randomized PBD for evaluating factors influencing ACP production by *B. sonorensis* strain ACP2

Std order	Trails	Variables													ACP productivity (U L^−1^ min^−1^)		
		*X*_*1*_	*X*_*2*_	*X*_*3*_	*X*_*4*_	*X*_*5*_	*X*_*6*_	*X*_*7*_		*X*_*8*_	*X*_*9*_		*X*_*10*_	*X*_*11*_	Actual value	Predicted value	Residual
9	1	1	1	1	-1	-1	-1	1		-1	1		1	-1	44.2039	44.90942	-0.7055
12	2	-1	-1	-1	-1	-1	-1	-1		-1	-1		-1	-1	31.6019	32.30742	-0.7055
17	3	0	0	0	0	0	0	0		0	0		0	0	39.7448	38.38223	1.3625
10	4	-1	1	1	1	-1	-1	-1		1	-1		1	1	34.4131	35.11864	-0.7055
15	5	0	0	0	0	0	0	0		0	0		0	0	39.8417	38.38223	1.4594
3	6	1	-1	1	1	-1	1	1		1	-1		-1	-1	51.4743	52.1798	-0.7055
5	7	-1	-1	1	-1	1	1	-1		1	1		1	-1	31.6019	32.30742	-0.7055
16	8	0	0	0	0	0	0	0		0	0		0	0	39.7448	38.38223	1.3625
1	9	1	1	-1	1	1	1	-1		-1	-1		1	-1	36.3519	37.05741	-0.7055
14	10	0	0	0	0	0	0	0		0	0		0	0	39.9386	38.38223	1.5564
4	11	-1	1	-1	1	1	-1	1		1	1		-1	-1	31.0203	31.72579	-0.7055
13	12	0	0	0	0	0	0	0		0	0		0	0	39.7448	38.38223	1.3625
8	13	1	1	-1	-1	-1	1	-1		1	1		-1	1	37.2244	37.92985	-0.7055
6	14	-1	-1	-1	1	-1	1	1		-1	1		1	1	30.2448	30.95029	-0.7055
2	15	-1	1	1	-1	1	1	1		-1	-1		-1	1	30.7295	31.43498	-0.7055
7	16	1	-1	-1	-1	1	-1	1		1	-1		1	1	55.8365	56.54203	-0.7055
11	17	1	-1	1	1	1	-1	-1		-1	1		-1	1	37.4182	38.12373	-0.7055
18	18	0	0	0	0	0	0	0		0	0		0	0	39.7448	38.38223	1.3625
Variable				Code		Coded and actual levels											
						-1			0			1					
Glucose				*X*_*1*_		1			5.5			10					
Na-glutamate				*X*_*2*_		0.1			0.3			0.5					
NH_4_NO_3_				*X*_*3*_		0.1			0.3			0.5					
NH_4_Cl				*X*_*4*_		0.1			0.3			0.5					
Eggshells				*X*_*5*_		0.2			1.1			2					
KCl				*X*_*6*_		0.1			0.3			0.5					
MgSO_4_.7H_2_O				*X*_*7*_		0.02			0.11			0.2					
NaH_2_PO_4_				*X*_*8*_		0.02			0.11			0.2					
(NH_4_)_2_MoO_4_				*X*_*9*_		0.0005			0.0015			0.0025					
CrCl_2_.6H_2_O				*X*_*10*_		0.0005			0.0015			0.0025					
FeSO_4_.7H_2_O				*X*_*11*_		0.0005			0.0015			0.0025					

$$Y={\beta }_{0}+\sum_{i=1}^{k}{\beta }_{i}{X}_{i}$$

The model intercepts are denoted by *β*_*o*_, the linear regression coefficient by *β*_*i*_, the coded independent variable estimates by *X*_*i*_, and *Y*'s expected response (ACP activity U L^−1^ min^−1^) [17]. To determine ACP productivity, variables with the uppermost *t*-values and confidence ranks of more than 95 percent (*p* < 0.05) were measured as significantly influential. This study's findings showed that independent variables such as *X*_*1*_, glucose; *X*_*7*_, MgSO_4_•7H_2_O; *X*_*8*_, NaH_2_PO_4_; and *X*_*10*_, CrCl_3_•6H_2_O had the most noticeable influence on ACP throughput; therefore, they were chosen by Orthogonal Central Composite Design (OCCD) to undergo further optimization. The average ACP production for the anticipated ideal values of the independent factors was evaluated and computed using a confirmatory experiment.

### Response surface methodology (OCCD)

After conducting PBD to screen independent factors and determine the ideal value of each independent variable that would provide the maximum ACP output, the four most impactful independent variables were selected for further optimization. To maximize the ACP output, ACP activity (*Y*) was taken from PBD as a dependent response parameter that needed to be enhanced. An OCCD was used to look at the correlation between four independent factors (glucose, *X*_*1*_; MgSO_4_•7H_2_O, *X*_*2*_; NaH_2_PO_4_, *X*_*3*_; and CrCl_3_•6H_2_O, *X*_*4*_) and one dependent variable (ACP activity, Y) at five distinct levels designated as (− α, − 1,0, + 1, + α).

The quadratic model with cross-product terms is the polynomial equation most usually used to represent the response surface. It is as follows:

$${Y=\beta }_{0}+\sum_{i}{\beta }_{i}{X}_{i}+\sum_{ii}{\beta }_{ii}{X}_{i}^{2}+\sum_{ij}{\beta }_{ij}{X}_{i}{X}_{j}$$

In this case, *X*_*i*_ and *X*_*j*_ are the independent variables, *β*_*i*_ represents linear coefficients, *β*_*ij*_ represents cross-product coefficients, and *β*_*ii*_ represents quadratic coefficients. *Y* is the expected response (ACP activity U L^−1^ min^−1^); *β*_*0*_ is the model intercept. A total of 36 trials with 16 cube points plus 12 middle points and 8.0 axial points were constructed using a 2^ k^-factor central composite design (Table 3) for additional improvement of the four medium parameters that contributed the most and significantly improved the productivity of *B. sonorensis* strain ACP2. A compatible analysis was performed on the ACP activity response data in these 36 combinations, and the regression coefficients and *t*-ratios versus the standard error were calculated. Statistical Designs, a free download program, had to be used to analyze each response value output. It produced reduced quadratic blended models for each response value at each time point. The analysis of variance (ANOVA) of each model revealed the primary components that had a statistically significant impact. Surface plots in three dimensions were created to aid in the display of the interaction between relevant factors and their ideal values using the STATISTICA software package. The average outcomes of each experiment, conducted in triplicate independently, are shown below.

### Scaling up the synthesis of bacterial ACP using bioprocessing approaches

By carefully stepping up the ACP manufacturing process from a shaking flask level to a bench-top fermenter scale, the current study sought to assess the growth dynamics of *B. sonorensis* strain ACP2 in an immersed cultivation system.

### Shake-flask batch cultivation

In 250-mL Erlenmeyer flasks comprising 50 mL of the antiseptic improved ACP throughput medium [(g L^−1^); glucose, 25.24; NH_4_NO_3_, 0.5; eggshell powder, 2; MgSO_4_•7H_2_O, 0.637; NaH_2_PO_4_, 0.455; CrCl_3_•6H_2_O, 0.0056; FeSO_4_•7H_2_O, 0.0015; without pH adjustment], shake-flask batch cultivation was carried out for 32 h at 45 ± 2 °C and 200 rpm in the study. The production medium was inoculated with a 5% (v/v) inoculum that was 12.0 h old. Bacterial culture samples were withdrawn for growth tracking every two hours throughout the incubation period by spectrophotometric monitoring at 600 nm. The samples were centrifuged at 10,000 rpm for 15 min at 4 °C to monitor various analytical parameters, such as ACP activity, glucose concentration, total soluble phosphate concentration, and total soluble protein concentration. All experiments were executed in triplicate.

### Batch cultivation system with stirred bioreactor

The microbial production process should be scaled up by optimizing growth from a shake flask to a fermenter. This investigation used the same inoculum size, temperature, and pH for all the stirred-tank reactor (STR) cultivations performed in shaking flasks with an optimized medium. A 7-L stirred tank reactor (STR; Bioflow 310, New Brunswick, NJ, USA) with a 4-L working capacity was employed, as reported by Abdelgalil et al. [15]; the procedure was automated using a 10.4 administrative panel color touch-screen PC system and bio-command multi-process monitoring program. 200 rpm was chosen as the agitation speed and kept constant during cultivation. Filtered sterile air was continuously used to aerate the system at a rate of 0.5 v/v/min. The foam was prevented from developing by using an antifoam agent (0.5% silicon oil). Polarographic electrodes for pH and DO were employed. Still, under a controlled pH culture setting, the pH was kept constant at 6.7 by a pH regulator passing on acid/base feeding peristaltic pumps connected to 2 M HCl and 2 M NaOH. 20 ml of culture samples were obtained at various points during the 32-h cultivation and placed in 50 ml sterile falcon tubes previously weighed. The Beckman DU spectrophotometer was utilized to display the light absorption at 600 nm compared to a blank, based on a track of cell proliferation. Centrifugation was performed for 15 min at 10,000 rpm to recover the cell-free supernatant, which was then used for additional analytical studies. The collected cell pellets were then utilized to assess the dry cell biomass, according to Abdelgalil et al. [10].

### Procedures for analysis

#### Glucose concentration determination

An enzymatic colourimetric kit measured glucose content in culture filtrate samples (Diamond Diagnostics, Egypt).

### Protein content and total soluble phosphate measurement

The ascorbate's and Lowry's procedures were employed to ascertain the bacterial culture filtrate's soluble phosphate and protein contents, respectively [18, 19].

### Morphological structure and energy-dispersive spectroscopy (EDS) analysis of the eggshell

The surface morphology of the obtained eggshell samples before and after fermentation was examined using a scanning electron microscope (Jeol JSM 6360 LA, Japan) linked to an in-situ energy-dispersive X-ray (EDS) spectrophotometer at the SRTA-city laboratory facility.

### Particle size analysis

Eggshell residue samples were collected, and their particle sizes were measured before and after fermentation using a particle size analyzer (PSA; Mod.: N5, Beckman Coulter, USA) at the SRTA-city laboratory facility.

### Detection of organic acid production

LC–MS/MS was used to detect and measure the organic acids produced by the strain under investigation. The organic acids were measured using a Luna® 3 µm HILIC column (100 × 4.6 mm) from Phenomenex in the United States. In mobile phase A, an HCOOH solution in water was used, whereas an HCOOH acid solution (mobile phase B) was used in methanol. Temperatures were maintained at 40 °C, and the column had a 20 µL capacity and was run at a flow rate of 0.35 mL min^−1^. As a reference, the 80:20 v/v main solution mixtures of the selected organic acids (1.0 mg mL^−1^) were made and stored at 4.0 °C for a month. A mixed intermediate stock solution of pyruvic acid (50 µg mL^−1^), lactic acid (10 µg mL^−1^), gluconic acid (1.5 µg mL^−1^), glutamic acid (1.2 µg mL^−1^) tartaric acid (1 µg mL^−1^), citric acid (0.5 µg mL^−1^), maleic acid (0.4 µg mL^−1^), salicylic acid (0.4 µg mL^−1^), and succinic acid (0.3 µg mL^−1^) was made using suitable dilutions using a water–methanol combination (80:20, v v^−1^). A series of serial dilutions were performed on the intermediate stock solution to establish standard working solutions that spanned the linearity ranges listed in Table S1. 20 µL of functional standard solutions were found using the recommended LC–MS/MS method. The quality control samples were made at three different concentration levels using the water–methanol mixture (80:20, v v^−1^): medium (MQC), high (HQC), and low (LQC), as presented in Table S2.

### SPE (solid-phase extraction) and sample cleanup

Particles from the samples were eliminated using a single-use, non-sterile cellulose syringe filter (0.45 µm). Four millilitres of 25 mM ammonium acetate were used to dilute an equal proportion of each sample filtrate, and 1% ammonium hydroxide was used to raise the pH to (6–7). As stated in the product manual, the SPE cartridge manufacturer's suggested methods were followed. Before loading the sample, the SPE pad (Strata X-AW, 200 mg 3 mL^−1^) was stabilized. 1.0 mL of methanol and 25 mM ammonium acetate (pH 6-7) were added for each of the three washes. Then, 1 mL of 5% ammonium hydroxide in methanol was used to elute the acids from the SPE cartridge. Using the proposed LC–MS/MS procedure, 20 µL of the reconstituted water–methanol combination (80:20, v v^−1^) was injected after the eluted solution had evaporated to dryness at 40 °C under a nitrogen stream.

### Software and instrumentation

An Eksigent ekspert™ ultraLC 100 system (Dublin, California, USA) comprising two connected pump units—one including an integrated degasser and the other featuring a mixer, chilled auto-sampler, and column oven chamber—was utilized to perform the UHPLC study. Mass spectrometric detection was achieved by multiple reaction monitoring (MRM) using the SCIEX QTRAP® 5500 (SCIEX instruments, Foster City, Canada). A Turbo VTM electrospray ionization (ESI) interface was used under negative ionization modes. The Analyst® software 1.6.2 was used to operate the mass spectrometer and UHPLC. Post-run data processing was carried out using MultiQuant 3.0. The samples were concentrated using a TurboVap® LV evaporator operated under nitrogen from Biotage GB Limited, UK.

### Quantitative MRM approach and chromatographic settings

Targeted organic acids were quantitatively monitored using an adverse MRM scanning strategy. An airtight syringe and a 7 µL/min flow rate were employed to inject the mass spectrometer with the organic acids of interest at 20 to 50 ng mL-1 concentrations. The optimum MRM transitions of the chosen organic acid and the crucial operating parameters of the mass spectrometer's ion source are displayed in Tables S3 and S4, respectively. Figures S1 through S10 display the targeted organic acids' ESI–MS/MS spectra and suggested fragmentation patterns. Table S5 provides a comprehensive overview of the chromatographic elution processes for the quantitative analysis.

### Method verification

The validation of the LC–MS/MS procedure was carried out by the most recent guidelines on bioanalytical method validation [20] and analytical technology validation [21] published by the International Conference on Harmonization (ICH).

### Atomic absorption analysis (AAS)

Using Zeenit 700 Analytik Jena, Germany, an AAS instrument, the heavy metal levels in the obtained eggshell samples were analyzed both earlier and afterward the cultivation process at the SRTA-City laboratory facility.

### Thermal analysis (TGA–DSC)

Thermal properties and pyrolysis pattern of the obtained eggshell samples were examined at the SRTA-city laboratory facility using a differential scan calorimeter (60–A) and a thermogravimetric detector (TGA, Model 50/50H, Shimadzu, Japan) both beforehand and following the fermentation process. In a nitrogen atmosphere with a flow rate of 20 mL min^−1^, TGA and DSC studies were carried out while the temperature was gradually raised from 10 to 700 °C at a steady heating rate of 10 °C min^−1^.

### Fourier-Transform Infrared Spectroscopy (FT-IR)

Using a Shimadzu FTIR-8400 S, Japan, at the SRTA-city laboratory facility, the active chemical bonds or functional groups linked to the obtained eggshell samples were investigated ahead of and following the cultivation process. KBr disk technique was employed as the matrix, and a 4 cm^−1^ mid-IR spectrum between 4000 and 400 cm^−1^ resolution was scanned.

### X-ray diffraction (XRD) analysis

At the Faculty of Science Laboratory Center, Alexandria University, the crystal structure, phase, and texture of the collected eggshell samples preceding and following the cultivation process were identified using an X-ray diffractometer (Bruker MeaSrv [D2-208219]) with CuKα (k = 1.54A°) radiation and scanned within a 2° to 100° 2θ angular range at a scan speed of 0.02°/s.

## Results

### Isolation and identification of ACP-producing bacteria

**Fig. 2:**
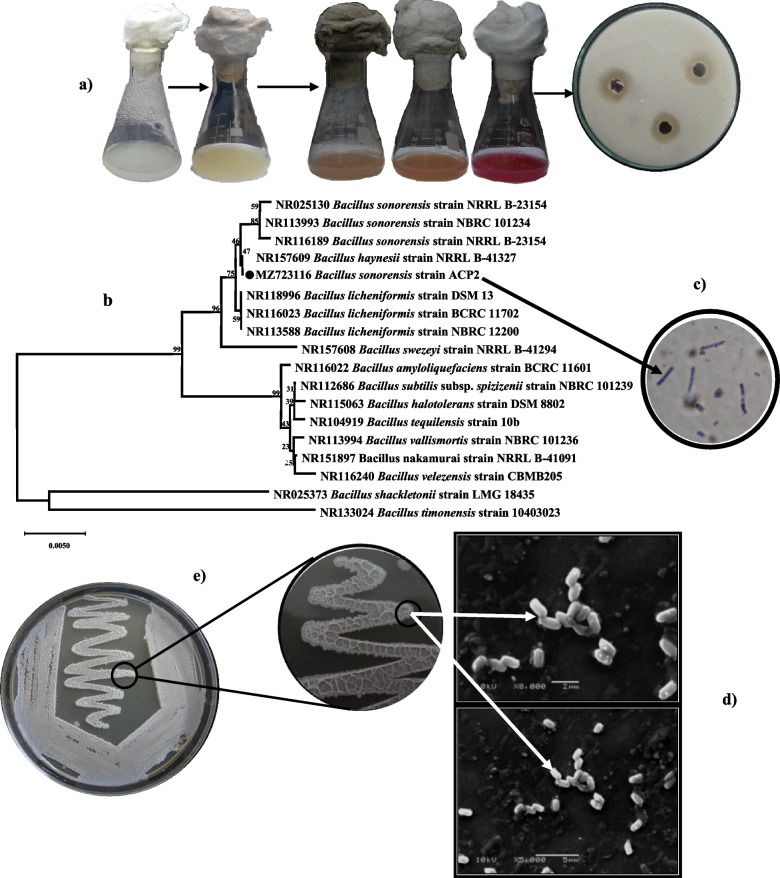
Isolation, screening, and identification of PSB and ACP-producing bacteria. **a** Isolation and qualitative screening of CaCO_3_-solubilizing and ACP-producing bacteria. **b** A phylogenetic tree based on *16S rDNA* gene sequence analysis shows the relationship of *B. sonorensis* strain ACP2 with reference strains (NCBI GenBank) constructed using the neighbor-joining method with the aid of the MEGA X program. **c** Gram-stain of the *B. sonorensis* strain ACP2 (using magnification, oil lens 100 x). **d** SEM micrograph of *B. sonorensis* strain ACP2 showing cell morphology at a magnification of 5000 and 10,000 × with 15 kv. **e** Cultural feature of *B. sonorensis* strain ACP2 agar plate

The program for isolation methods utilized sewage samples from the paper and pulp mills in Alexandria, and 20 distinct morphotypes of isolates were obtained. The ACP-producing bacteria with organic acid-producing potentialities were identified and picked up according to the halo zone development on PKV agar medium and the production of a pink-coloured product in culture broth upon cleavage of an artificial chromogenic substrate as shown in Fig. 2a. On PKV's plates, the isolate entitled ACP2 demonstrated the furthermost apparent clusters with a noticeable clear zone, as well as the highest obviousness of purple colour intensity in PDP-LB culture broth, and the most increased ACP activity of 39 U L^−1^ min^−1^ amongst the chosen isolates shown in Fig. 2a. Based on this data, it is reasonable to conclude that the most potent chosen isolate has varied organic acids producing efficacy. As a result, the ACP2 isolate was selected for further investigation. Prior to sequencing, the *16S rRNA* gene was extracted from genomic DNA and purified. The *16S rRNA* array of ACP2 isolates yielded a nucleotide sequence of 1334 bp, which was determined and then submitted to a BLAST search in the GenBank database. ACP2 was shown to have 100% sequence identities to *B. sonorensis*, *B. licheniformis*, *B. haynesii*, *and B. paralicheniformis*, indicating that it was genetically linked to members of the *Bacillus* genus. In addition, the strain's total and maximum score of 2464 was recorded, and the analysis showed that it had 100% query coverage for all related species. Accession number MZ723116 has been allocated to the strain by GenBank.

Further confirmation was provided by the neighbour-joining dendrogram tree, which revealed that the strain is phylogenetically in the clade of the genus *Bacillus* and formed distinct groups with other species of the genus *Bacillus*, as shown in Fig. 2b. Phylogenetic analysis based on *16S rRNA* gene sequence analysis indicated that the ACP2 strain is identified in a clade with *B. sonorensis* NRRL B-23154 T; this phyletic track, jointly with *B. licheniformis* BCRC 11702, was constantly detected in the same clade as *B. sonorensis* strain. Despite having a low bootstrap value (47%), the ACP2 strain was shown to cluster always with *B. haynesii* NRRL B-41327, as determined by various treeing approaches, and *B. swezeyi* NRRLB-41294 formed a distinct clade with it. Based on taxonomic criteria, the strain ACP2 was given the suggested name *B. sonorensis* strain ACP2 since it closely resembled *B. sonorensis*. During the microscopical description of strain ACP2, spore-forming bacteria and rod-shaped bacteria measuring within 1.2 to 1.6 µm in height and 0.73 to 0.86 µm in diameter were found (Fig. 2c and d). Cells with movable and catalase-positive characteristics are often found alone, while they can occasionally be seen in chains of two to four cells. Amorphous and mucous slime is formed in the form of mounds or lobes on LB agar by colonies that are white, irregularly shaped, and have a rough, wrinkled-like labyrinth pattern, textured surface with ridges and furrows as illustrated in Fig. 2e.

### Conventional physical parameter optimization for ACP synthesis

According to the results of this investigation, the *B. sonorensis* strain ACP2 produced the highest amount of ACP (39.14 U L^−1^) when grown at 45ºC. Transport of substances across the cells is inhibited at temperatures below the optimum (37ºC), and a smaller amount of enzymes is obtained (34.4 U L^−1^). Additionally, the activated pre-cultures were kept at 5% (v/v) inoculum size to get the highest ACP output (41.8 U L^−1^); ACP throughput was felled down to 38.8 U L^−1^ when 10% of the inoculum volume was utilized. Moreover, the ACP output trend steadily increases from pH 3 (36.35 U L^−1^) to pH 7 (43.11 U L^−1^) before decreasing to 35.9 U L^−1^ at pH 8; it seems that the isolated bacterium is mostly neutrophilic and requires an ideal pH of 7.0 for growth and ACP productivity. When it comes to maximum ACP production (46.9 U L^−1^), however, compared to the adjusted pH medium with 1N HCl, it appears that the unchanged pH medium (approximately pH 6.7–6.9) exhibits the best and optimal circumstances (43.11 U L^−1^). As a result, a pH-unadjusted medium was employed in all subsequent experiments conducted as part of this work.

### Statistical tuning of *B. sonorensis* strain ACP2's ACP synthesis

Using the PBD, an established experimental design matrix of eighteen trials was created to prescreen the impact of eleven distinct medium ingredients, namely glucose, sodium glutamate, NH_4_NO_3_, NH_4_Cl, eggshell powder, KCl, MgSO_4_•7H_2_O, NaH_2_PO_4_, ammonium molybdate, CrCl_3_•6H_2_O, FeSO_4_•7H_2_O, corresponding to *X*_*1*_*–X*_*11*_, at their bottommost and uppermost factor points on ACP production. Table 1 exhibits that ACP's productivity varies widely across the design array, underscoring the necessity of medium improvement in boosting ACP efficacy. Both glucose (*X*_*1*_) and NaH_2_PO_4_ (*X*_*8*_) are required for the induction and synthesis of ACP; the fact that trials number 6 and 16 had the highest ACP activity (~ 51.4–55.8 U L^−1^) and had high amounts of both nutritive media (10 and 0.2 g L^−1^, respectively) shows this as presented in Table 1. Additionally, as a result of the use of the lowest concentrations of glucose and NaH_2_PO_4_ (1 and 0.02 g L^−1^, respectively), the ACP throughput decreased by ~ 30.2–30.7 U L^−1^ as seen in trial numbers 14 and 15, respectively. This finding, in turn, highlighted and emphasized the value of glucose and NaH_2_PO_4_ in strengthening ACP production.

### Results of the mathematical multiple regression study of PBD

**Fig. 3:**
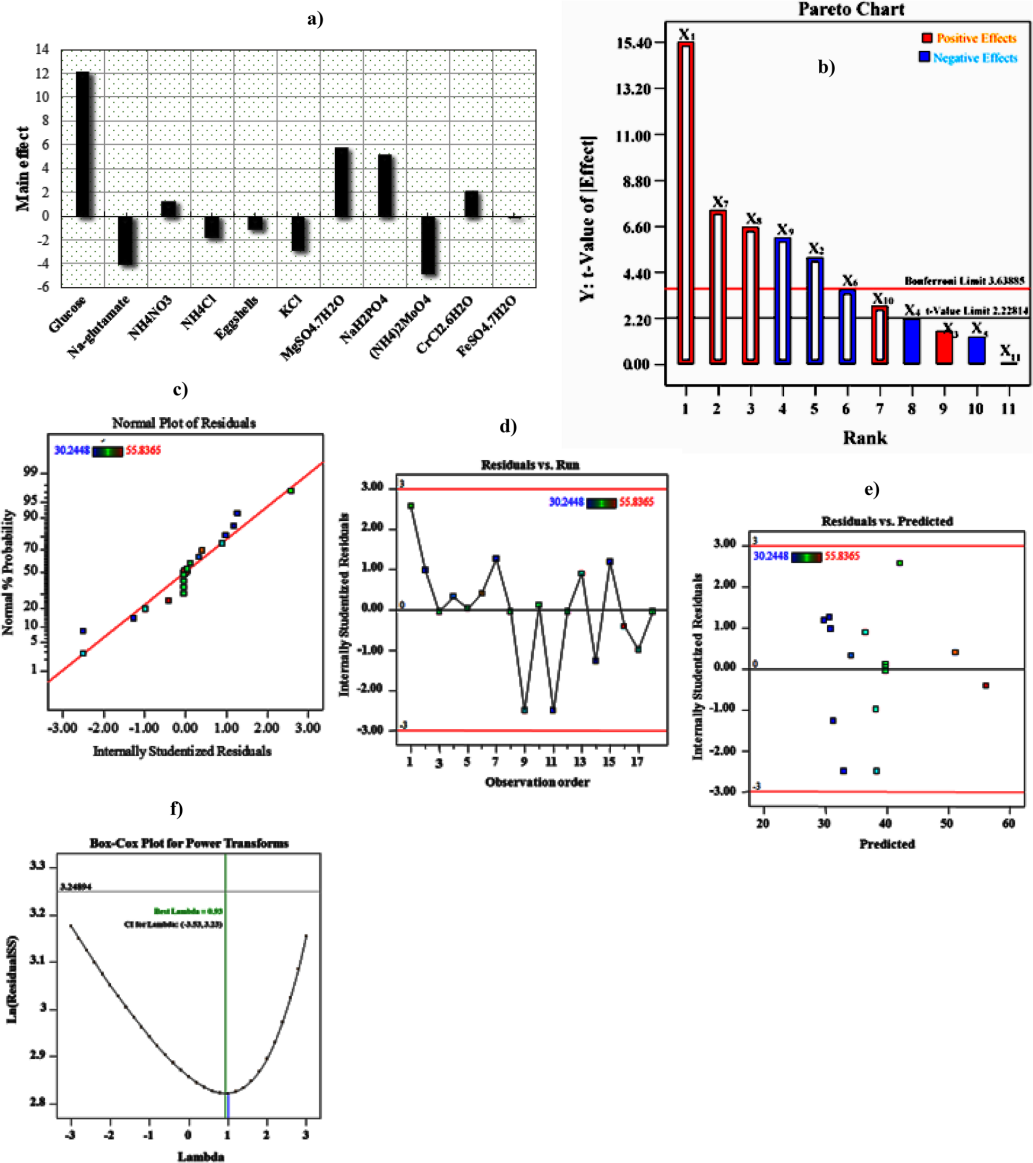
PBD results: **a** Main effect of culture variables. **b** Pareto chart illustrating the order and significance of the variables affecting ACP production by *B. sonorensis* strain ACP2. **c** The first-order polynomial equation determines the standard probability plot of the residuals for ACP production. **d** Correlation between the residual and observation order. **e** Correlation between the residual and predicted values(**f**) Box-Cox plot

The obtained coefficients and the *t*- and *p*-values are provided in Table 2. It is effective for identifying substantial main effects but not significant interactions between factors due to the design's peculiarities. Table 2 and Fig. 3a depict the significant impacts of all independent variables on ACP production. As a strategy for developing optimal settings for ACP output, it was appropriate to consider the sign (+ or -) and the degree of the main effect. The results demonstrated that the glucose followed by MgSO_4_•7H_2_O, NaH_2_PO_4_, and CrCl_3_•6H_2_O demonstrated a noteworthy increase in ACP output, suggesting that the highest concentration of these parameters is needed for optimal production of ACP. Sodium glutamate, ammonium molybdate, KCl, and NH_4_Cl_2_ significantly affected ACP synthesis, whereas FeSO_4_•7H_2_O had a negligible influence. However, the eggshell powder should be kept to a minimum for enhanced ACP output. The ANOVA findings also support this since the *p*-value of 1.88 E^−05^ and *t*-value of 12.16 indicates that glucose (*X*_*1*_) substantially influences the production process (a contribution percentage of 29.6); this is evident in Table 2. MgSO_4_•7H_2_O (*X*_*7*_) and NaH_2_PO_4_ (*X*_*8*_) both exhibited confidence levels of 99.88 and 99.79 percent, respectively; they also had a considerable influence on ACP production with a* t*-value of 5.82, 5.17, a *p*-value of 0.0011, 0.0020, and an involvement percentage of 14.17 and 12.59; respectively. On the other hand, CrCl_3_•6H_2_O was statistically significant at 92.99 percent with a *p*-value of 0.07, *t*-value of 2.2, and contribution percent of 5.35; it also substantially influenced ACP production. There was no noticeable effect on ACP yield from the other coefficient components included in this model.

**Table 2:** Statistical analysis of PBD showing coefficient values, *t*- and *p*-values for each variable affecting ACP production

Variables	Coefficient	Main effect	Std Error	*t*-Stat	*P*-value	Contribution %	Confidence level (%)
Intercept	38.3822		0.40769	94.1451	9.68E^−11^		100
X_1_	6.0748	12.1496	0.49931	12.1661	1.88E^−05^	29.6063	99.9981
X_2_	-2.0195	-4.0391	0.49931	-4.0446	0.00676	9.84252	99.3232
X_3_	0.6301	1.2602	0.49931	1.26191	0.25381	3.070866	74.6186
X_4_	-0.8562	-1.7125	0.49931	-1.7149	0.13718	4.173228	86.2815
X_5_	-0.517	-1.0340	0.49931	-1.0354	0.34038	2.519685	65.9610
X_6_	-1.4056	-2.8112	0.49931	-2.8150	0.03055	6.850394	96.9447
X_7_	2.9081	5.8163	0.49931	5.8242	0.00112	14.17323	99.8873
X_8_	2.5850	5.1700	0.49931	5.1771	0.00206	12.59843	99.7939
X_9_	-2.3911	-4.7823	0.49931	-4.7888	0.00303	11.65354	99.6964
X_10_	1.0986	2.1972	0.49931	2.2002	0.07007	5.354331	92.9923
X_11_	-0.0323	-0.0646	0.49931	-0.0647	0.95050	0.15748	4.94958
**ANOVA**	**Df**	**SS**	**MS**	***F***	**Significance *F***		
**Regression**	11	797.046	72.45873	24.21885	0.0004	**Std. Dev**	1.37
**Residual**	6	17.95099	2.991832			**Mean**	38.38
***R***^***2***^	0.9789					**C.V.%**	3.56
**Adj. *R***^***2***^	0.9625					**PRESS**	151.10
**Pred. *R***^***2***^	0.8104					**Adeq. Precision**	27.2797

Figure 3b indicates that effects above the Bonferroni limit are likely to be significant, effects above the *t*-value limits are possibly significant (and should be evaluated if they haven't already been chosen), and the Pareto diagram indicates that effects below the *t*-value limits are unlikely to be significant. The glucose (*X*_*1*_), MgSO_4_•7H_2_O (*X*_*7*_), and NaH_2_PO_4_ (*X*_*8*_) effects cross the Bonferroni limit; that is, it showed a significant influence on the ACP production. Figure 3c depicts the typical plot of the standardized impact of adequate nutrients, representing the greatness and guidance of their powerful impacts. It was noticed that near the center (zero) line, most effects cluster around the fitted standard model straight line [22]. The residuals are shown against the experimental run order, as illustrated in Fig. 3d. Furthermore, the constant variance assumption may be confirmed since the residuals in Fig. 3e are oriented against the expected response. The experimental runs' points were all randomly given. All values fell between 3 and -3, indicating that the models suggested by the PBD were appropriate and that the constant variance assumptions were reasonable. Figure 3f shows the smallest values and lambda, representing the 95 percent confidence interval. The findings in Table 2 demonstrate that the present model can account for almost 90% of the response variation. It was discovered that the correlation coefficient (*R*^*2*^) was 97.89 percent. A 95 percent confidence level was achieved for each regression model, and the *F*-value was found to have a very high value (24.2) with a low probability (0.0004), indicating that the regression model is statistically significant. This means that 97.89 percent of the experimental data were consistent with the model, with only 2.11 percent of the variation not explained by the model. It also reveals a strong relationship between model importance, the investigated variables, and ACP production, given the high adjusted coefficient of determination value (Adj. *R*^*2*^ = 0.9625).

#### Regression equation

According to the results of the ANOVA, the first-order model describing the relationship between the eleven factors evaluated over 18 tryouts and the ACP yield may be stated as the following equation:

$${Y}_{activity}= 38.38+6.07{X}_{1}-2.019{X}_{2}+0.630{X}_{3}-0.856{X}_{4}-0.517{X}_{5}-1.405{X}_{6}+2.908{X}_{7}+2.585{X}_{8}-2.391{X}_{9}+1.098{X}_{10}-0.032{X}_{11}$$

The Plackett–Burman design's findings were confirmed by conducting a validation experiment. At 45°C and 200 rpm, the ideal culture conditions for the production of ACP were estimated to be (g L^−1^): glucose, 10; NH_4_NO_3_, 0.5; eggshell powder, 0.2; MgSO_4_•7H_2_O, 0.2; NaH_2_PO_4_, 0.2; CrCl_3_•6H_2_O, 0.0025; FeSO_4_•7H_2_O, 0.0015, and 5% activated inoculum volume for 24 h incubation time. The maximal ACP activity was 72.4 U L^−1^, more significant than the activity obtained before the PBD was employed (43.11 U L^−1^) by 1.67 fold.

### R*esponse surface methodology (RSM)*

The glucose, MgSO_4_•7H_2_O, NaH_2_PO_4_, and CrCl_3_•6H_2_O dimensionless coded variables (with confidence levels of 99.99, 96.88, 99.79, and 92.9%, respectively) investigated via the preceding PBD were subjected to further examination to assess their interaction and the modelling process, the RSM model was constructed using an empirical half-factor OCCD. Thirty-six experiments were done utilizing a precision random array of eight axial, sixteen factorials, and twelve midpoints by the OCCD. For the selected variables, consideration was given to parameter symbols, values, the layout of the array, and actual and anticipated outcomes (all displayed in Table 3). It was shown that the efficiency of ACP activity varied significantly based on the four parameters described above. It is shown in Table 3 that at the axial level of the NaH_2_PO_4_, with the minimal concentration of 0.15 g L^−1^ in experimental trials (number 22), 204.44 U L^−1^ of ACP was produced (predicted to be 188.71). Alternatively, run 31 produced the most negligible ACP activity of 128 U L^−1^ when the other predictors were kept at zero, except glucose, which was supplied at its minimal axial level (-2) at an amount of 8 g L^−1^. An important finding from this study shows that glucose was needed to stimulate ACP synthesis by the *B. sonorensis* strain ACP2.

**Table 3:** Matrix designed for *B. sonorensis* strain ACP2 OCCD

Std order	Trials		Variables						ACP productivity (U L^-1^ min^-1^)		
		Type	*X*_*1*_	*X*_*2*_		*X*_*3*_		*X*_*4*_	Actual value	Predicted value	Residual
19	1	Center	0	0		0		0	190.48	190.459	0.024
18	2	Factorial	-1	-1		1		1	140.75	143.832	-3.077
21	3	Center	0	0		0		0	191.06	190.459	0.605
14	4	Factorial	-1	1		1		-1	151.80	143.396	8.409
11	5	Factorial	-1	1		1		1	150.06	148.558	1.502
28	6	Factorial	-1	-1		-1		-1	157.62	164.528	-6.906
3	7	Factorial	1	1		1		-1	162.27	160.384	1.890
32	8	Center	0	0		0		0	190.48	190.459	0.024
4	9	Factorial	1	1		-1		-1	168.96	177.082	-8.118
16	10	Axial	0	2		0		0	174.48	173.689	0.7997
24	11	Factorial	1	-1		-1		1	129.12	148.727	-19.60
6	12	Center	0	0		0		0	190.19	190.459	-0.266
12	13	Center	0	0		0		0	190.77	190.459	0.315
17	14	Axial	0	0		2		0	158.20	166.516	-8.312
30	15	Center	0	0		0		0	189.90	190.459	-0.557
25	16	Axial	0	0		0		2	153.25	148.388	4.871
34	17	Factorial	1	-1		1		1	161.69	152.387	9.306
15	18	Center	0	0		0		0	190.77	190.459	0.315
27	19 2	Center	0	0		0		0	190.19	190.459	-0.266
36	20	Factorial	-1	-1		1		-1	146.28	141.869	4.410
26	21	Factorial	1	-1		-1		-1	156.16	153.889	2.278
20	22	Axial	0	0		-2		0	204.44	188.714	15.728
35	23	Factorial	-1	-1		-1		1	151.22	149.333	1.8902
5	24	Center	0	0		0		0	190.48	190.459	0.0242
22	25	Axial	0	-2		0		0	152.38	145.771	6.6160
13	26	Axial	2	0		0		0	158.20	147.855	10.348
7	27	Factorial	1	-1		1		-1	131.44	140.391	-8.942
9	28	Axial	0	0		0		-2	150.93	148.388	2.544
23	29	Factorial	-1	1		-1		-1	163.72	169.254	-5.525
10	30	Center	0	0		0		0	189.90	190.459	-0.557
1	31	Axial	-2	0		0		0	128.54	131.472	-2.932
29	32	Center	0	0		0		0	190.48	190.459	0.024
31	33	Center	0	0		0		0	190.77	190.459	0.315
8	34	Factorial	1	1		1		1	171.29	175.579	-4.289
2	35	Factorial	-1	1		-1		1	155.00	157.258	-2.253
33	36	Factorial	1	1		-1		1	174.48	175.119	-0.630
Variables		Code	Coded and actual levels								
			-2		-1		0		1	2	
Glucose			8		16		24		32	40	
MgSO_4_.7H_2_O			0.18		0.36		0.54		0.72	0.9	
NaH_2_PO_4_			0.15		0.45		0.75		1.05	1.35	
CrCl_2_.6H_2_O			0.002		0.004		0.006		0.008	0.01	

### ANOVA and multiple regression analysis

The analysis and interpretation of the CCD experimental data results were done using multiple regression statistical analysis and ANOVA calculations, which are essential techniques for assessing the significance and practicality of the quadratic regression model (Table 4). Only 8.2% of the total difference generated by variables could not be described by this model, nor could ACP activities be explained, according to the ANOVA, which indicated a determination coefficient *R*^*2*^ of 0.912, meaning that the model equation could explain 91.2 percent of the whole variance in the data. They were using an adj. *R*^*2*^ of 0.854, the model's relevance and precision, and the little coefficient of change (CV = 4.77 percent) of a tryout's data can be shown. According to the model, a signal-to-noise ratio of 11.43 indicates a sufficient signal to ensure the model can move through the design space. According to Fisher's *F*-test of 15.73 and 4.48E^−08^, the model is entirely meaningful with the slightest standard deviations (7.99), as demonstrated by the mean unbiased residuals to mean square regression ratio. It is calculated that the ACP productivity model has a residual error number of squares (PRESS) value of 7720. As a result of the results presented in Table 4, the positive coefficients for the linear effects of the variables *X*_*1*_, *X*_*2*_, and *X*_*4*_, as well as the positive coefficients for the mutual effects of the variables *X*_*1*_*X*_*2*_, *X*_*1*_*X*_*3*_, *X*_*1*_*X*_*4*_, *X*_*2*_*X*_*4*_, and *X*_*3*_*X*_*4*_ show that these variables have synergistic effects that increase the production of the ACP. This is supported by the data on their* F*-values, *p*-values, *t*-values, and contribution percent. However, the antagonistic impact was seen in the linear (*X*_*3*_) and mutual interaction (*X*_*2*_*X*_*3*_), as well as the quadratic effects (*X*_*1*_^*2*^, *X*_*2*_^*2*^, *X*_*3*_^*2*^, and *X*_*4*_^*2*^) of the variables under investigation. It was determined that they had made no substantial contribution to boosting ACP production by *B. sonorensis* strain ACP2, based on the negative sign of their coefficients and computational indications for their significance degrees. Regression coefficients were used to fit an equation with a second-order polynomial (Table 4). ACP production (*Y*) by *B. sonorensis* strain ACP2 may be stated as a regression equation as follows:

**Table 4:** ANOVA for the response surface of ACP production by *B. sonorensis* strain ACP2 obtained by OCCD. “Std. Dev. is the standard deviation, the coefficient of determination (*R*^*2*^), Adj *R*^*2*^ is the adjusted-*R*^*2*^, and PRESS is the prediction error sum of squares, C.V is the Coefficient of variation”

Term	Coefficient Estimate	Mean square	*t*-Stat	*F*-value	*p*-value	Confidence level (%)	Contribution (%)
Intercept	190.45	1005	82.550	15.73	7.15E^−28^		
*X*_*1*_	4.0956	402.58	2.5104	6.30	0.0203	97.968	6.201834
*X*_*2*_	6.9795	1169	4.278	18.30	0.0003	99.966	10.56880
*X*_*3*_	-5.5497	739.19	-3.401	11.57	0.0026	99.731	8.403669
*X*_*4*_	3.23E^−15^	0	1.9E^−15^	0	1	0	4.88424E^−15^
*X*_1_*X_*2*_	4.6166	341.02	2.310	5.34	0.0311	96.889	6.9908256
*X*_1_*X_*3*_	2.2901	83.92	1.146	1.31	0.2646	73.538	3.4678899
*X*_1_*X_*4*_	2.5082	100.66	1.255	1.58	0.2231	77.685	3.7981651
*X*_2_*X_*3*_	-0.79974	10.23	-0.40	0.1602	0.6930	30.698	1.2110091
*X*_2_*X_*4*_	0.79974	10.23	0.400	0.1602	0.6930	30.698	1.2110091
*X*_3_*X_*4*_	4.2895	294.40	2.146	4.61	0.0436	95.635	6.4954128
*X*_1_*X_*1*_	-12.698	5160	-8.988	80.79	1.21E^−08^	100	19.229357
*X*_2_*X_*2*_	-7.6823	1888	-5.437	29.57	2.15E^−05^	99.997	11.633027
*X*_3_*X_*3*_	-3.2110	329.95	-2.272	5.17	0.03366	96.633	4.8623853
*X*_4_*X_*4*_	-10.517	3539	-7.444	55.42	2.56E^−07^	99.999	15.92660
	**df**	**SS**	**MS**	***F***	**Significance *F***		
**Regression**	14	14,070	1005	15.733	4.48E^−08^	**Std. Dev**	7.99
**Residual**	21	1341	63.87			**Mean**	167.72
**Total**	35	15,411				**C.V. %**	4.77
***R***^***2***^	0.912					**PRESS**	7720
**Adj. *R***^***2***^	0.854						
**Adeq. Precision**	11.43						

$${Y}_{activity }=190.45+4.095{X}_{1}+6.979{X}_{2}-5.549{X}_{3}+3.23{E}^{-15}{X}_{4}+4.616{X}_{1}{X}_{2}+2.290{X}_{1}{X}_{3}+2.508{X}_{1}{X}_{4}-0.799{X}_{2}{X}_{3}+0.799{X}_{2}{X}_{4}+4.289{X}_{3}{X}_{4}-12.69{X}_{1}^{2}-7.68{X}_{2}^{2}-3.21{X}_{3}^{2}-10.51{X}_{4}^{2}$$

In this case, the independent variables glucose, MgSO_4_.7H_2_O, NaH_2_PO_4_, and CrCl_3_.6H_2_O are coded at values *X*_*1*_, *X*_*2*_, *X*_*3*,_ and *X*_*4*_, respectively, and *Y* represents the expected response (ACP activity).

### Model suitability assessment

**Fig. 4:**
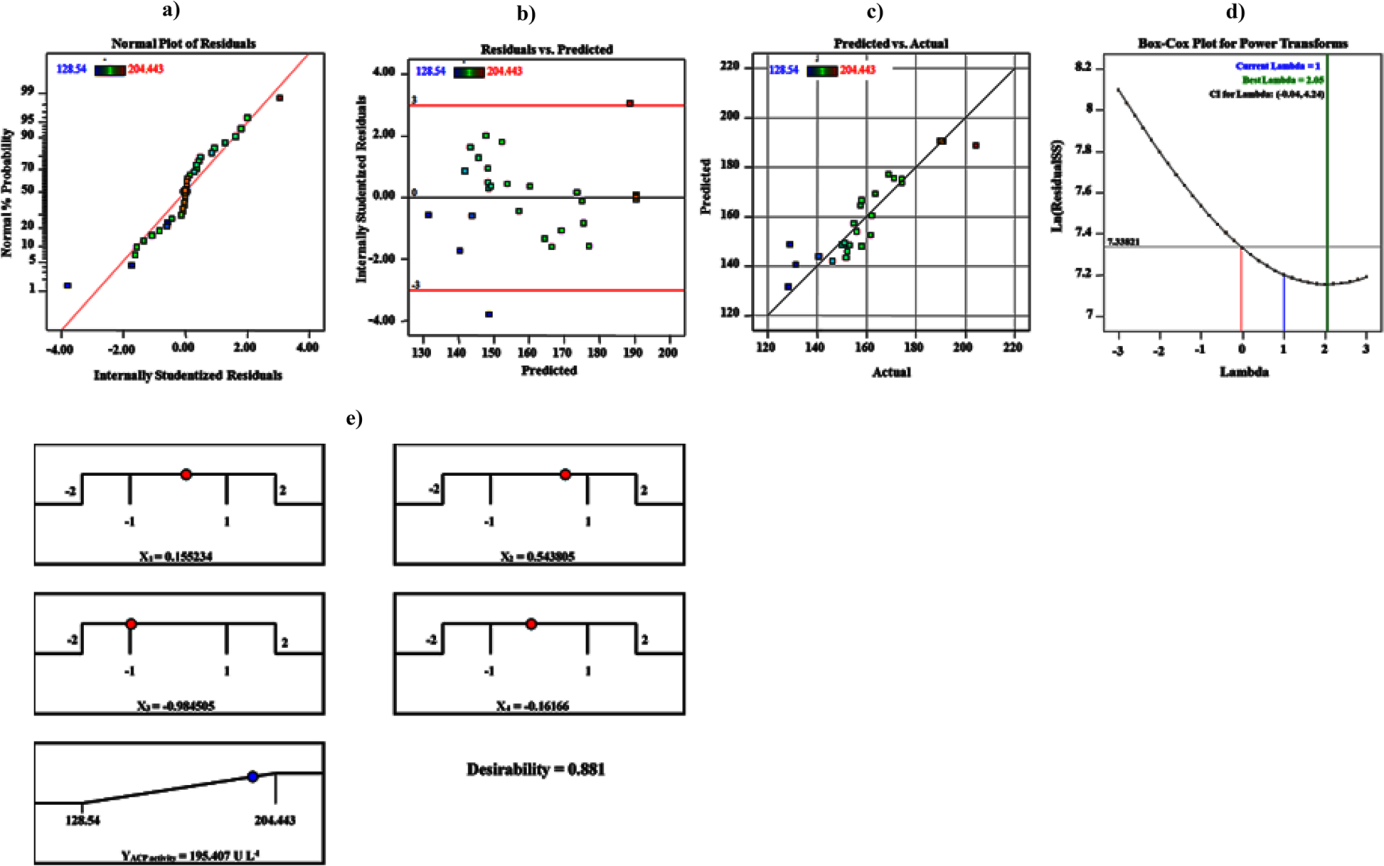
Model adequacy checking of OCCD: **a** Normal probability plot of the residuals. **b** Externally studentized residuals versus predicted ACP production. **c** Plot of predicted versus actual ACP production. **d** Box-Cox plot. **e** The optimization plot displays the desirability function and the optimum predicted values for the maximum ACP production

A typical linear distribution for the residuals is shown by the compact grouping of data points along the straight line, as seen in Fig. 4a. As illustrated in Fig. 4b; the residuals were drawn versus their anticipated response, which confirmed the assumption of constant variance. Furthermore, the uniform distribution of the data points across a 45-degree line is shown in Fig. 4c. All the data points may be obtained, showing the model's validity. Figure 4d provides a better understanding of the Box-Cox graph. The green line represented the best *λ*-value (2.05), the blue line represented the transformation (*λ* = 1), and the lines in red represented the confidence intervals' lowest and most significant values (-0.04 and 4.24, respectively). Consequently, these model diagnostic charts showed that the fitted model for the response met its assumptions and that, on the whole, the model matched the data well.

### Optimization using the desirability function (DF)

Experimental design aims to attain the conditions that will provide the best results by predicting the optimum ones. The desirability function and an optimization plot with the most significant predicted values for the ideal production of ACP are shown in Fig. 4e. At 45°C and 200 rpm for 24 h, the ACP2 strain of *B. sonorensis* reported the maximum foreseen value of ACP (195.45 U L^−1^) in the existence of g L^−1^: glucose (25.24), NH_4_NO_3_ (0.5), eggshell powder (2), MgSO_4_•7H_2_O (0.637), NaH_2_PO_4_ (0.455), CrCl_3_•6H_2_O (0.0056) and FeSO_4_•7H_2_O (0.0015) without pH adjustment. The experimental findings and predicted values were fully promising, indicating that the DF could accurately compute the ideal anticipated conditions for ACP synthesis by *B. sonorensis* with an accuracy of almost 99%.

### Three-dimensional (3D) plots and contours

**Fig. 5:**
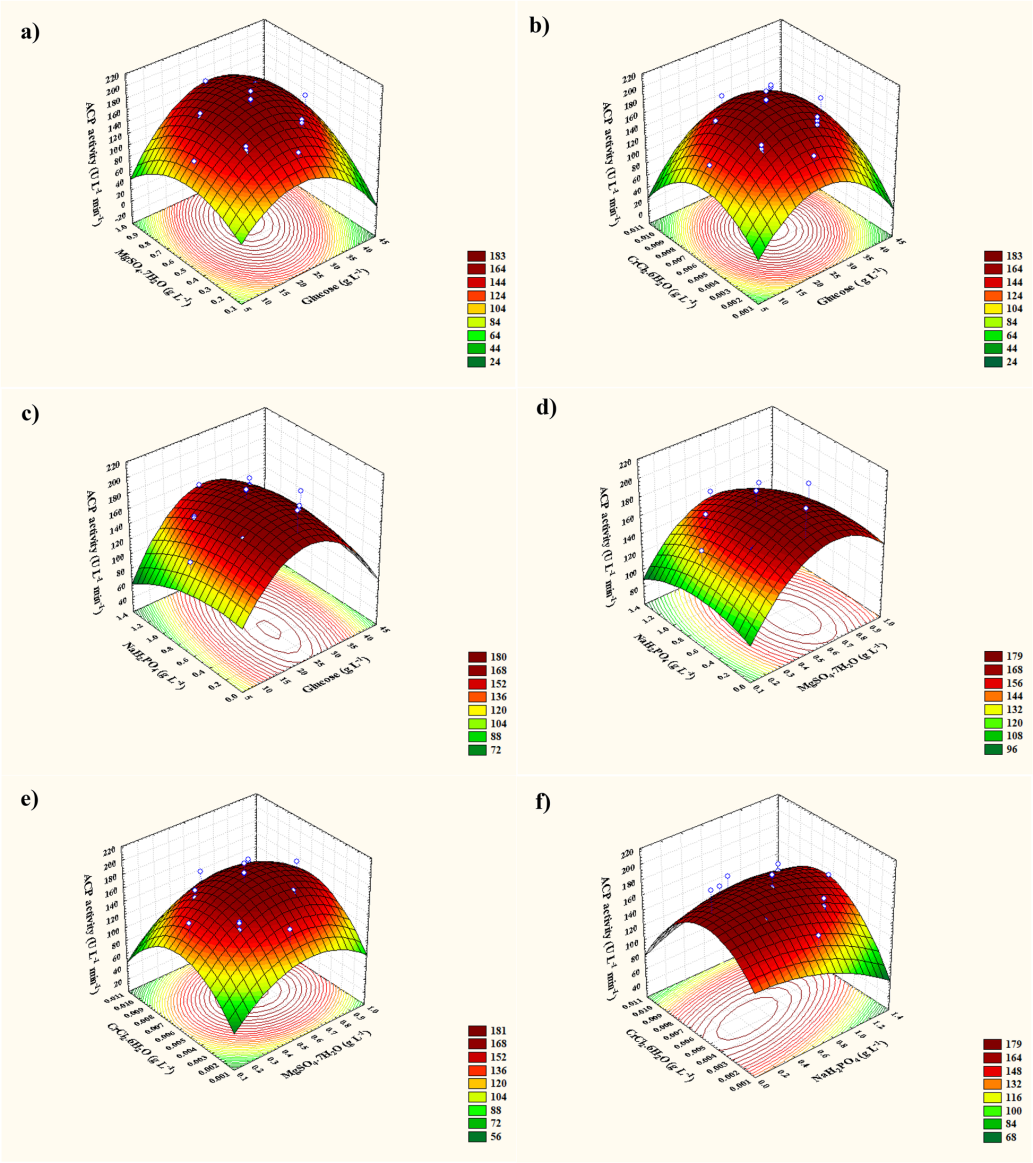
3D response surface representing ACP activity yield (U L^−1^ min^−^.^1^) from *B. sonorensis* strain ACP2 as affected by culture conditions: **a** Interaction between MgSO_4_.7H_2_O and glucose, **b** Interaction between CrCl_3_.6H_2_O and glucose, **c** Interaction between NaH_2_PO_4_ and glucose, **d** Interaction between NaH_2_PO_4_ and MgSO_4_.7H_2_O, **e** Interaction between CrCl_3_.6H_2_O and MgSO_4_.7H_2_O, and **f** Interaction between CrCl_3_.6H_2_O and NaH_2_PO_4_

The three-dimensional plots have established two independent factors of the four variables (glucose, MgSO_4_•7H_2_O, NaH_2_PO_4_, and CrCl_3_•6H_2_O) and ACP activity (U L^−1^). Figure 5 illustrates this by charting the ACP activity on the Z-axis versus each of them while keeping all other variables at zero. It was feasible to show how glucose and MgSO_4_.7H_2_O affect ACP synthesis simultaneously using a 3D surface map (Fig. 5a). ACP activity was at its greatest near of the glucose center point. In contrast, outside of this region, ACP production was insignificant. MgSO_4_•7H_2_O boosted ACP activity to a maximum near the MgSO_4_•7H_2_O center points, although a greater level of MgSO_4_•7H_2_O supporting low ACP activity was seen. 192.84 U L^−1^ of predicted ACP activity was achieved at the optimum projected glucose (26 g L^−1^) and MgSO_4_•7H_2_O (0.6357g L^−1^) at zero level of 0.75 and 0.006g L^−1^ for NaH_2_PO_4_, and CrCl_3_•6H_2_O, respectively. A comparable trend in ACP effectiveness was observed regarding the other paired arrangement of the factors under study.

Furthermore, as shown in Fig. 5b, lower and higher glucose (*X*_*1*_) and CrCl_3_•6H_2_O (*X*_*4*_) concentrations are linked to decreased ACP activity. There is a clear correlation between the highest ACP activity (190.79 U L^−1^) and both glucose (*X*_*1*_) and CrCl_3_•6H_2_O's (*X*_*3*_) central point. This finding highlights the importance of glucose (*X*_*1*_) and CrCl_3_•6H_2_O (*X*_*3*_) in producing ACP. According to Fig. 5c, increasing NaH_2_PO_4_ (*X*_*3*_) to 0.499 g L^−1^ and glucose (*X*_*1*_) to nearly its center point level (24.688 g L^−1^) caused ACP production to peak (192.94 U L^−1^), demonstrating how closely the process of synthesis depends on the starting concentrations of glucose and NaH_2_PO_4_, with the two other variables maintained at zero values. 3D and contour plots (Fig. 5d) showed that MgSO_4_•7H_2_O (*X*_*2*_) and NaH_2_PO_4_ (*X*_*3*_) affected ACP production at 24 g L^−1^ of zero-level glucose (*X*_*1*_) concentration and 0.006 g L^−1^ of zero-level CrCl_3_•6H_2_O (*X*_*4*_). When NaH_2_PO_4_ (*X*_*3*_) concentrations were dropped to near their axial point (0.471 g L^−1^), followed by a rise in MgSO_4_•7H_2_O (*X*_*2*_) concentrations to their optimum (0.63 g L^−1^), ACP activity steadily increased; after that, ACP output dropped. The maximum ACP throughput (194.78 U L^−1^) was supported at the center level of MgSO_4_•7H_2_O (*X*_*2*_), crossing the ideal points of its concentration; any change therein would result in a loss of productivity. Figure 5e shows that increasing MgSO_4_•7H_2_O (*X*_*2*_) concentration slightly beyond its center point (0.621 g L^−1^) boosted ACP production (192.048182 U L^−1^) when CrCl_3_•6H_2_O (*X*_*4*_) was present at or near its mid-point (0.006 g L^−1^) while maintaining glucose (*X*_*1*_) and NaH_2_PO_4_ (*X*_*3*_) at their zero levels. Conversely, rising both concentrations over their recorded points led to a decline in ACP throughput.

On the other hand, it was noticed that when using glucose (*X*_*1*_) and MgSO_4_•7H_2_O (*X*_*2*_) held at their zero levels (Fig. 5f), the ACP production efficiencies rose linearly with increasing CrCl_3_•6H_2_O (*X*_*4*_) concentration, accomplishing the pinnacle of efficiency (193.23 U L^−1^) at the CrCl_3_•6H_2_O (*X*_*4*_) center point (0.0059 g L^−1^). Furthermore, when the concentration of CrCl_3_•6H_2_O continues to rise, the production of ACP decreases stepwise. However, the lowest level of NaH_2_PO_4_ (*X*_*3*_) (0.449 g L^−1^) was found near its axial point, which helped to promote the production process. Despite this, Fig. 5f plot analysis shows that the above and below the optimum recorded point for NaH_2_PO_4_ (*X*_*3*_) concentrations yielded little ACP production. The findings showed that none of the factors had a substantial relationship with each other or with boosting the production of ACP overall, as indicated by the three-dimensional (3D) contour plots.

### Scale-up fermentation approaches for enhancing ACP production

For ACP's industrial-scale commercialization, an engineering perspective and a scaling-up approach were both established as part of the effort to increase scientific understanding.

### Shake-flask batch conditions: cell growth kinetics and ACP production

To ascertain whether there is a relationship between the rate of ACP output and a culture's specific growth, the production of extracellular ACP was monitored during the growth of *B. sonorensis* strain ACP2 on a boosted medium in a shake flask under ideal cultivation settings. Table 5 demonstrates the effects of various cultivation approaches on the *B. sonorensis* strain ACP2's proliferation dynamics and ACP synthesis properties.

**Table 5:** Kinetic parameters of cell growth and ACP production by *B. sonorensis* strain ACP2 as affected by different cultivation strategies. *X*_*max*._, maximal cell dry weight; $\frac{dX}{dt}$, cell growth rate; µ, specific growth rate; *P*_*max*_, maximal ACP production; *P*_*max specific*_, specific productivity; ${Q}_{p}$, ACP production rate; ${Q}_{s}$, substrate consumption rate; ${Y}_\frac{p}{x}$, U g^−1^ of ACP produced per g biomass; ${Y}_\frac{p}{s}$ U g^−1^ of ACP produced per g substrate consumed; ${Y}_\frac{x}{s}$ g g^−1^, of biomass produced per g substrate consumed

Parameters	Shake flask cultivation	Uncontrollable pH Batch Cultivation	Controllable pH Batch Cultivation
Growth parameters			
*X*_*max*_ (g. L^−1^)	5.986	6.016	6.559
µ (h^−1^)	0.090	0.1	0.085
µ_Max_ (h^−1^)	0.583	0.634	1.090
$\hspace{1em}\frac{dX}{dt}$(g. L^−1^. h^−1^)	0.315	0.374	0.396
Production parameters			
*P*_*max*_ (U L^−1^)	194.26	216.37	64.416
*P*_*max.specific*_ (U g^−1^)	134.21	182.98	51.389
*P*_*max.time*_ (h)	28	18	26
$\hspace{1em}{Q}_{p}$(U L^−1^ h^−1^)	5.023	7.888	1.5146
-${Q}_{s}$ (g. L^−1^. h^−1^)	0.678	1.13	0.4
Yield coefficient parameters			
$\hspace{1em}{Y}_\frac{p}{s}$(U g^−1^)	4.89	5.12	3
$\hspace{1em}{Y}_\frac{p}{x}$(U g^−1^)	13.73	18.2	5.13
$\hspace{1em}{Y}_\frac{x}{s}$(g g^−1^)	0.19	0.27	0.39
Overall cultivation time (h)	32	32	32

**Fig. 6:**
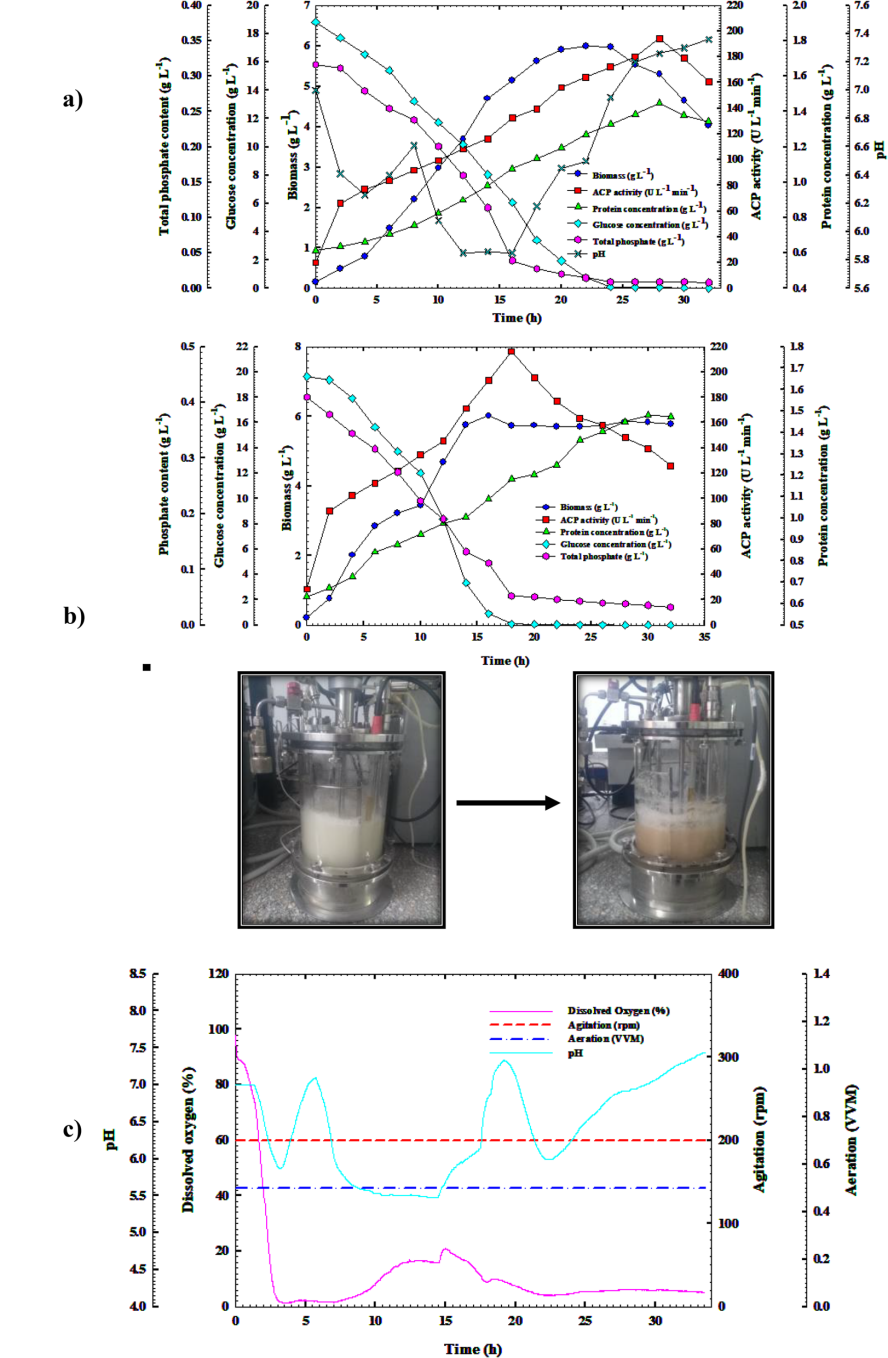
Monitoring of *B. sonorensis* strain ACP2 growth and ACP productivity in (**a**) A shake-flask scale cultivation condition. **b** A 7 L stirred-tank bioreactor under uncontrolled pH conditions. **c** Online data (DO, agitation, aeration, and pH) as a function of time during batch fermentation in the bioreactor under uncontrolled pH conditions

Figure 6a shows that the ACP2 strain of *B. sonorensis* appropriately developed, and ACP was formed simultaneously with cell growth. Cells grew exponentially subsequent to a lag time, with a growth rate of 0.315 (g L^−1^ h^−1^) and a specific growth rate (*µ*) of 0.098 h^−1^. Biomass production (5.98 g L^−1^) reached its zenith at 22 h of the cultivation time with a noticeable yield coefficient *Y*_*x/s*_ (0.19 g g^−1^), propelling cell growth to the stationary phase. It was found that the expression of the genes encoding acid phosphatase was progressive from the commencement of the cultivation to its apex (194.26 U L^−1^), at which point the throughput rate (*Q*_*p*_) was 5.023 U L^−1^ h^−1^. This elevation occurred late in the stationary phase (28 h). The cells have more time to synthesize ACP early in the culture phase since they grow more slowly. It was found that the ACP's yield coefficients *Y*_*p/s*_ and ACP’s specific productivity *P*_*max*_ were (4.89 and 134.21 U g^−1^, respectively). The protein concentration trends also showed these findings; it crested (1.44 g L^−1^) at the end of the *B. sonorensis* strain ACP2 stationary phase, while ACP production peaked (at 28 h). Since then, ACP activity and protein quantity have declined over time. With a mean consumption rate of 0.678 g L^−1^ h^−1^, the glucose concentration dropped from its beginning level of 18.82 g L^−1^ at the beginning of the time of cultivation to barely 0.019 g L^−1^ at the end. This decline was caused by the proliferation of cells and ACP's critical role in the transportation and utilization of phosphate. In the meantime, the quantity of phosphate dropped sharply from the beginning level of 0.315 g L^–1^ to 0.008 g L^–1^ at the end of the time of cultivation due to ACP's critical role in the transportation and utilization of phosphate, suggesting the vital function of phosphate for the proliferation of bacteria and the process of ACP output.

A drop in pH was seen in Fig. 6a due to the CaCO_3_ solubilization process, which was associated with producing organic acids, resulting in a gradual decline in the pH of *B. sonorensis* strain ACP2 cultural medium from 6.45 to 5.85 during the first 16 h of cultivation, followed by a gradual rise back to pH 7.36 at the end of the cultivation. Incremental bacterial growth, ACP throughput, and protein quantity increases coincided with the pH change. This result was caused by how pH affects the development of bacteria or how pH regulates the expression of genes that produce enzymes, as explained by Qureshi et al. [23].

### Heavy metals survey

AAS was used to examine and identify heavy metals in the eggshell residues from the shake-flask cultivation. Leftover eggshell samples from the shake-flask cultivation process showed that excessive amounts of K^+^, Fe^+^, Cd^2+^, Cr^+^, Zn^+^, Mn^2+^, Sn^+^, Ag^+^, and Cu^2+^ were solubilized and accumulated during the fermentation process, resulting in an increase in their concentrations by a factor of 4.437, 2.162, 1.157, 11.367, 1.765, 2.383, 89.166, 150.65, and 2.806; respectively, when compared to their early amounts beforehand fermentation at zero time as cited in Table 6. The findings indicate that during the *B. sonorensis* strain ACP2's solubilization process, the metals Ag^+^, Sn^+^, and Cr^+^ were most effectively liberated from eggshells. The growth of *B. sonorensis* strain ACP2, on the other hand, was associated with a drop in concentrations of cobalt, nickel, magnesium, and lithium as well as calcium and sodium during the fermentation process, by factors of 89.8, 1.82, 1.72, 1.747, 1.89 and 1.91; respectively compared to those noticed in an eggshell leftover sample at zero time incubation. This result emphasizes the critical role that these metal ions play in the proliferation and ACP synthesis.

**Table 6:** Flame AAS for heavy metals analysis of eggshells powder leftover samples during shake-flask cultivation system at zero incubation time and after 32 h of the cultivation process

Metals conc. (mg/Kg)	Eggshells leftover samples at zero incubation time	Eggshells leftover samples after 32 h
Co	8.98	< 0.1
K	2478	**10,996.1**
Fe	73	**157.88**
Ni	3.732	2.05
Cd	1.0904	**1.262**
Cr	3.84	**43.65**
Zn	9.468	**16.72**
Mg	22,620	13,136.53
Mn	4.824	**11.496**
Li	2.312	1.323
Ca	580,440	306,000
Sn	< 1.2	**107**
Ag	2.336	**351.92**
Na	75,900	39,675
Cu	10.8	**30.31**

### Cell growth kinetics and ACP production in the bioreactor under uncontrolled pH batch conditions

*B. sonorensis* strain ACP2 was cultured in a bench-top 7 L stirred-tank fermenter with uncontrolled pH to facilitate further optimization and boost ACP production. It was evident from Fig. 6b that every pattern noticed in this investigation was quite comparable to the patterns established in its shake-flask equivalent. The top production rate *Q*_*p*_ (7.888 U L^−1^ h^−1^) and maximal ACP production, with the ACP specific productivity (182.98 U g^−1^), were detected as 216.37 U L^−1^ over the beginning of the stationary period (18 h) of *B. sonorensis* strain ACP2 growth after that, the production curve began to flatten down. The findings obtained were 1.11 times greater in production capability, 1.36 times higher in specific productivity, and 1.57 times faster in production rate than those published for the shake flask model. These results were followed by significant increases of 1.32 and 1.04 over those observed in shake flask mode in the ACP yield coefficients *Y*_*p/x*_ and *Y*_*p/s*_, which were 18.2 and 5.12 U g ^−1^, respectively.

Furthermore, there was a tendency for the protein content to increase along with the increase in ACP synthesis gradually; the protein concentration peaked at 1.47 g L^−1^ and then steadily increased until 28 h later. *B. sonorensis* strain ACP2 growth patterns in a shaking flask and a stirred-reservoir fermenter are strikingly similar. At 16 h of incubation, or roughly 6.0 h earlier than in the shake-flask, the most significant cell yield of biomass (*X*_*max*_; 6.016 g L^−1^) with a rate of proliferation of 0.374 g L^−1^ h^−1^ and a specific growth rate (µ) of 0.1 h^−1^ occurred. After surpassing its apex point, the stationary stage started at 18 h of incubation. According to the results, the obtained output coefficient *Y*_*x/s*_ of 0.27 g g^−1^ was marginally better than that from the shaking flask by a factor of 0.08. The amount of glucose in the culture media, tracked during the whole cultivation timing, was inversely correlated with ACP2 cell growth and ACP production. Cell growth and ACP production rose 1.66 times compared to shake-flask culture, resulting in a consumption rate (*Q*_*s*_) of 1.13 g L^–1^ h^–1^.

By the end of the growth phase at 28 h, the amount of glucose had been entirely consumed, in contrast to the starting glucose concentration of 19.65 g L^−1^. By the time the shake flask culture system reached the end of its cultivation cycle, the glucose amount had consumed 0.019 g L^−1^. Additionally, as shown in Fig. 6b, ACP2 cell growth and ACP production were accompanied by the intake of organic and inorganic phosphate media components. This finding resulted in a sharp decline in the level of soluble phosphate at the end of the growing duration, from the starting level of 0.409 to 0.0317 g L^–1^. The culture medium's pH pattern matched that of the corresponding shaking flask. While in the early log phase, pH values decreased from 6.94 to 5.68 after 4.0 h of incubation, then increased to 7.2 after 7.0 h, then dropped to 5.84 once more after 18 h, and finally stayed steady with some fluctuations until the completion of the cultivation, with pH values in the range of 6.0–6.34. It is believed that the unregulated pH culture condition contributed to improved ACP production. When the *B. sonorensis* strain ACP2 grew and increased in biomass rapidly during the early stages of fermentation, the resulting rapid fall in dissolved oxygen (DO) content was observed.

Consequently, the amount of DO in the growth medium was evaluated during this study's cultivation period. As the culture progresses into the stationary phase, the air supply may be curtailed, and the pH of the medium may be lowered, both of which may cause stress to the bacterial cell. As illustrated in Fig. 6c, when the DO concentration dropped to 0.9%, the ACP production began to increase gradually, reaching its peak at 18 h of cultivation time when the DO level was 6%; once this point was passed, the ACP production began to decline, and the DO concentration increased to 24% at the end of the cultivation time. A preliminary study found that phosphatase production increased when an organism was moved to anaerobiosis, which is compatible with the results of this study. The PhoP/PhoQ sensor/regulator system seems to have a role in promoting acid phosphatase production in the presence of stress, as discovered in an early study [24].

### Cell growth dynamics and the synthesis of ACP in the bioreactor with regulated pH batch settings

**Fig. 7:**
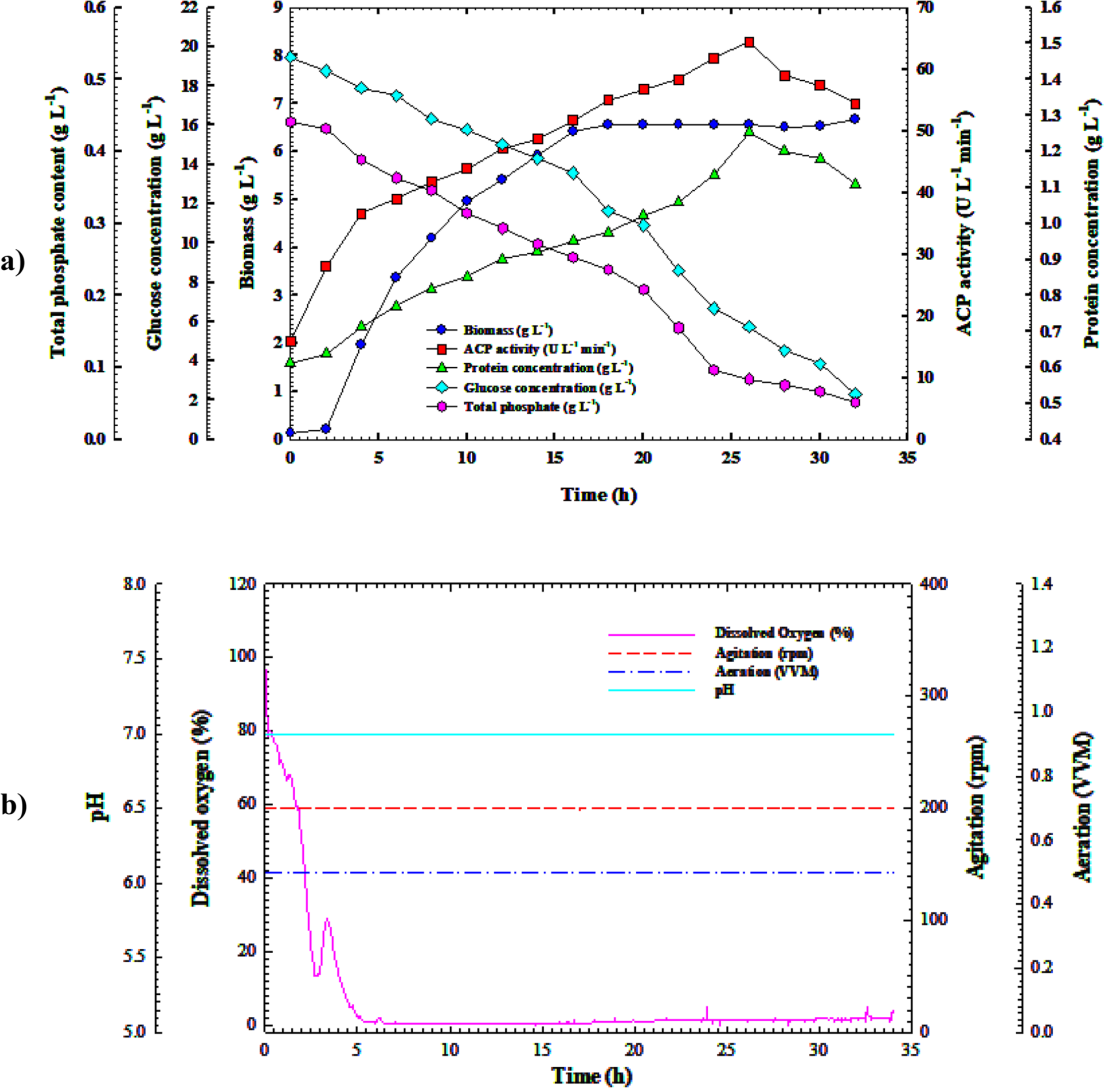
**a** Monitoring of *B. sonorensis* strain ACP2 growth and ACP productivity in a 7 L stirred-tank bioreactor under controlled pH conditions. **b** Online data (DO, agitation, aeration, and pH) as a function of time during batch fermentation

Furthermore, to get a deeper comprehension of the influence of pH on the production process, *B. sonorensis* strain ACP2 was cultivated in a 7-L bench-top bioreactor with regulated pH settings. The trends of substrate intake, volumetric ACP synthesis, and proliferation of cells that came from the study are shown in Fig. 7a. Similar to other methods of culture; there was no discernible phase lag in the exponential growth of the bacterial cells over time. The cell growth increased gradually over 18 h with a growth rate of 0.396 g L^1^ h^1^ and a specific growth rate (*µ*) of 0.085 h^−1^, reaching its maximum (*X*_*max*_ = 6.559 g h L^−1^) at 18 h. Although the growth duration was comparable to that of uncontrolled pH culture circumstances, this study showed a considerable increase in growth rate over shake-flask cultivation methods. However, the highest biomass production rose by 9.57 and 9%, respectively, over the shake-flask and uncontrolled pH culture approaches. Because of this, an increase in the yield coefficient *Y*_*x/s*_ of 0.39 g g^−1^ was observed, which was significantly more significant than the yield coefficients attained from the shaking flask and uncontrolled pH cultivation modes by two and one-fourth of a factor, respectively. While ACP2 growth was better than under uncontrolled pH cultivation conditions, ACP productivity was 10.0 h behind that of a wild pH system, where ACP volumetric productivity rose steadily throughout the cultivation period with a production rate (*Q*_*p*_) of 1.51, peaking at 64.41 U L^–1^ at 28 h. Comparing ACP2 cultivation in a shake flask and unadjusted pH conditions, the volumetric yield of ACP descended by 66.84% and 70.22%, respectively, which causes a decrease in the output coefficient *Y*_*p/x*_ of the biomass (5.13 U g^–1^) by 62.63, and 71.81% lower than that obtained through shake-flask and uncontrolled pH systems, respectively. In addition to the preceding, under the above culture condition, a 38.65 and 41.40% reduction in yield coefficient *Y*_*p/s*_ of 3 U g^−1^ was reported compared to earlier cultivation modes, respectively. The protein content trend followed the ACP production pattern and peaked at 1.253 g L^−1^. At the same time, ACP production peaked at 28 h, allowing for ACP's specific productivity (51.389 U g^–1^). After this, protein content and ACP activity showed a noticeable gradual decline. It was demonstrated that there was a correlation between substrate consumption and growth as well as ACP production. The levels of glucose and total phosphate went down from their starting levels (19.48 and 0.441 g L^−1^) to their minimal levels (2.316 and 0.0522 g L^−1^, respectively) towards the end of the 32-h growing period, with an average glucose consumption rate of 0.4 g L^−1^ h^−1^. This decline was because the consumption of nutrients increased in tandem with the proliferation of cells and the synthesis of enzymes. The glucose intake rate showed that, compared to the shake-flask and unregulated pH batch culture approaches, there was a 41 and 64.60% decrease in glucose consumption, respectively. Additionally, as exhibited in Fig. 7b, the percentage of DO decreased dramatically when the bacterial cells approached the exponential phase, reaching 1.6% at 4.0 h of cultivation time due to excessive oxygen consumption. Afterwards, the DO content fluctuated slightly, increasing again, reaching 21% after 15 h of incubation, before decreasing progressively, reaching 5% at the end of the incubation period, which coincided with ACP peak production.

#### Morphological structure of the eggshell

**Fig. 8:**
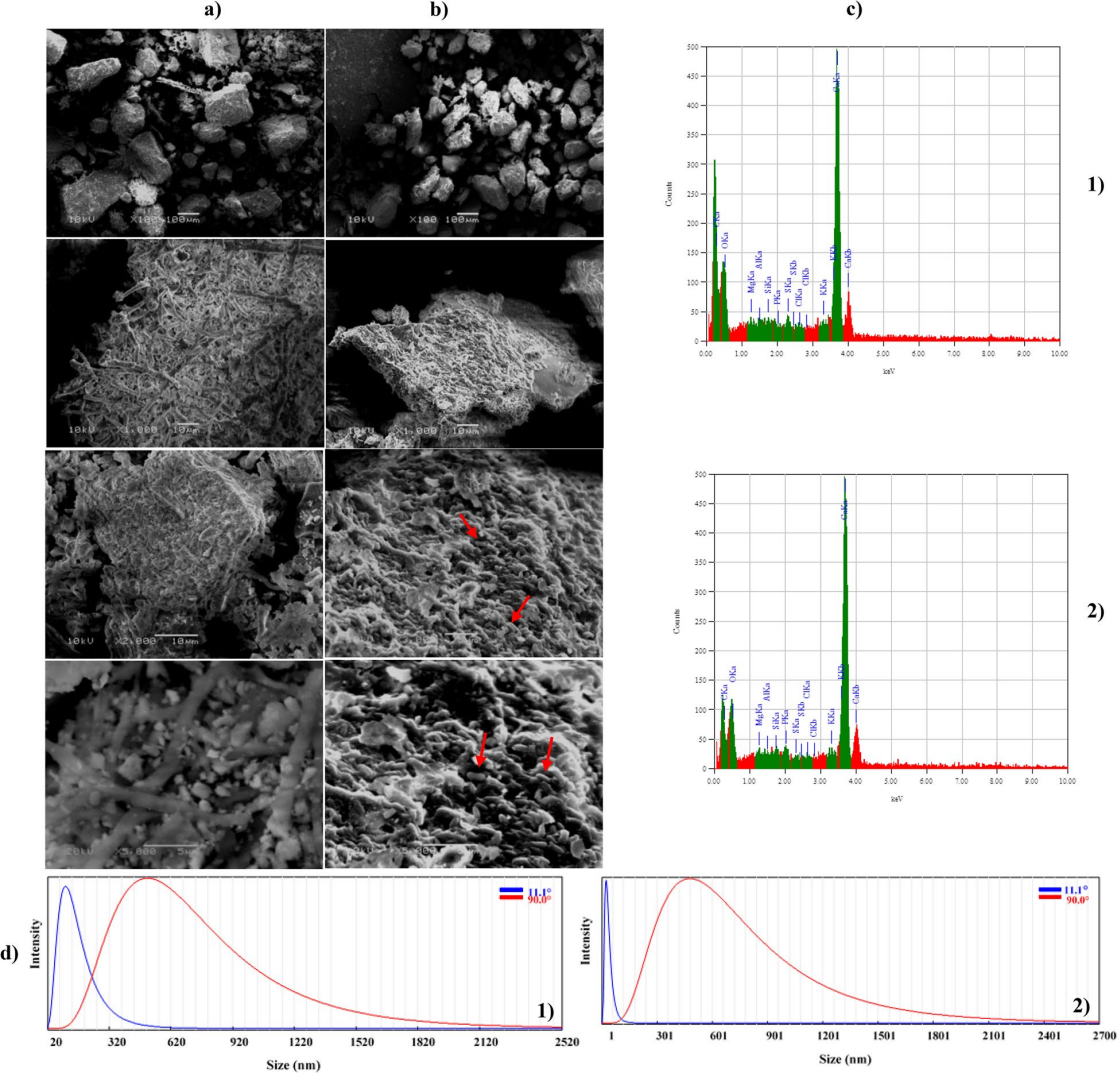
SEM micrograph at a magnification of 100x, 1000, 2000x, and 5000 × for the eggshells leftover of **a**) zero incubation time; **b** After 32 h of incubation time. **c** EDX analysis. **d** Particle size distribution analysis

Following the completion of fermentation, the residual eggshell powder samples were taken out, weighed, and dried in an oven for a whole night at 60 °C. SEM and EDS analyses were then performed on the collected eggshell powder to investigate the samples' dispersion and surface texture. Throughout the cultivation process, *B. sonorensis* strain ACP2 solubilized eggshell powder samples, resulting in an alteration in the obtained sample's appearance and metal element composition, as seen in Fig. 8. It can be shown in the SEM micrograph (Fig. 8a) that during zero-time incubation, the eggshell particles had a rough surface with uneven shapes and sizes and a fibre-like network structure encasing CaCO_3_ particles, which can be seen in the SEM micrograph (100x, 1000x, 2000x, and 5000x). According to Fig. 8c1, the eggshell particle's EDS indicates that its components are calcium (Ca), phosphorus (P), silica (Si), aluminium (Al), magnesia (Mg), oxygen (O), sulfur (S), Chlorine (Cl), potassium (K), and carbon (C). The maxima of absorption associated with Ca (AT% of 45.25 and mass % of 67.88), O (AT% of 34.77 and mass % of 20.82), and C (AT% of 16.64 and mass % of 7.48) are among the most prevalent in the sample. P is associated with the least prominent absorption peak (AT% of 0.14 and mass % of 0.17). Calcite (CaCO_3_) is the most abundant component in eggshell particles, confirming that calcium carbonate is the most abundant component. Thus, the eggshell hydroxyapatite's Ca: P weight ratio was 323.

On the other side, powdered eggshell morphology changed after 32 h of fermentation time, as shown in Fig. 8b. Bacterial cells were securely attached to the eggshell particles, which had smaller, looser-structured particles with irregular shapes and defined limits that vanished with a softening of the surface edges, as seen at a magnification of 2000 and 5000X, in Fig. 8b. However, the element map Fig. 8c2 shows a significant concentration of Ca (AT% of 68.40 and mass % of 82.34) followed by O (AT% of 22.70 and mass % of 10.91), and C (AT% of 2.71 and mass % of 0.98), as well as modest traces of Si, S, Cl, Mg, K, and P. In addition, Al was not detected in the sample by EDX analysis. The dissolution of CaCO_3_ due to the influence of organic acids and ACP synthesized by *B. sonorensis* strain ACP2 throughout the fermentation process was found to be the cause of the notable 51% increase in the AT% for Ca in comparison with those at zero time of incubation. The AT% for C and O reduced by 83.71% and 34.71, respectively, compared to those recorded before the cultivation process because it was consumed by bacterial cells for growth and metabolite production. Eggshell particles were solubilized by ACP, resulting in a 12.357-fold increase in P absorption peaks identified by EDS investigation (AT% of 1.73 and mass % of 1.61) compared to those observed at nil cultivation time.

As a result, the hydroxyapatite ratio by weight (Ca: P) of the eggshell was 39.537, 18.63% less than the ratio recorded preceding the cultivation. Furthermore, particular minerals and other soluble elements were liberated and leached from the powdery eggshell with the help of bio-solubilization and metabolic processes approaches. An arithmetical mean particle size study at different angles (90° and 11.1°) indicated that after 32 h of cultivation, the particle size of the eggshell (537.7 nm and 26 nm) had decreased by 2.57 and 78.13%, respectively, in comparison to the initial particle size (551.9 and 118.9 nm, respectively) (Fig. 8d1-Fig. 8d2).

### Organic acids production profile

**Fig. 9:**
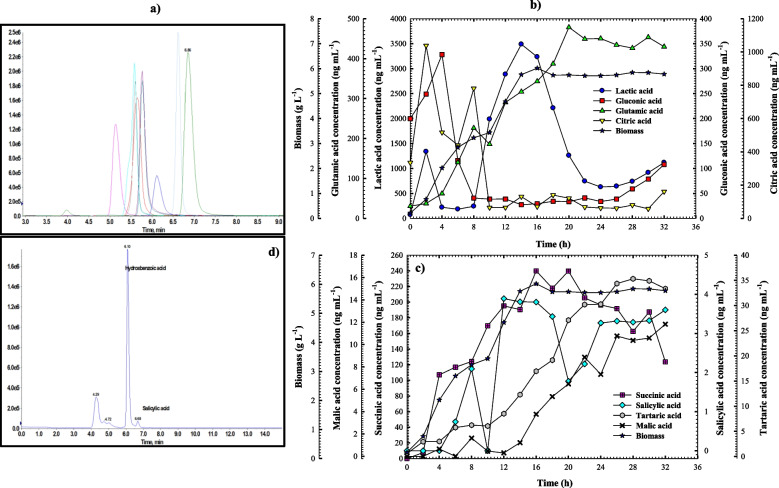
**a** Representative extracted negative ion MRM chromatograms of organic acids at the MQC: glutamic acid (*m/z*: 145.96/128.0, RT: 5.13 min), gluconic acid (*m/z*: 194.95/129.1, RT: 5.59 min), succinic acid (*m/z*: 116.88/72.9, RT: 5.60 min), lactic acid (*m/z*: 88.62/43.0, RT: 5.61 min), citric acid (*m/z*: 190.92/111.0, RT: 5.75 min), tartaric acid (*m/z*: 148.69/87, RT: 5.77 min), pyruvic acid (*m/z*: 86.94/43.0, RT: 6.12 min), salicylic acid (*m/z*: 136.94/93.0, RT: 6.63 min), and maleic acid (*m/z*: 114.91/71.0, RT: 6.85 min). **b** Monitoring of biomass, lactic, gluconic, glutamic, and citric acids production; **c** Monitoring of biomass, succinic, salicylic, malic, and tartaric acids production throughout shake-flask cultivation system; **d** Representative extracted negative ion MRM chromatograms illustrating the presence of hydroxybenzoic acid alongside salicylic acid

For organic acid detection, LC–MS/MS was used to evaluate *B. sonorensis* strain ACP2 uncontrolled pH cultivation broth at various incubation times for lactic acid, citric acid, maleic acid, salicylic, succinic acid, and so on. All detected organic acids are shown in Fig. 9a as MRM chromatograms extracted using negative ions as a standard. Of the eight unique organic acids that were tracked, lactic acid was found to be the most prevalent (3488 ng mL^−1^), followed by citric acid (1038.4 ng mL^−1^), glutamic acid (478.4 ng mL^−1^), gluconic acid (328.10 ng mL^−1^), succinic acids (239.70 ng mL^−1^), tartaric acid (34.36 ng mL^−1^), malic acid (10.765 ng mL^−1^), and the least prevalent kind of acid was salicylic (3.888 ng mL^−1^). Monitoring the organic acid production over the cultivation period demonstrated that, as Fig. 9b illustrates, lactic acid production was in phase with the proliferation of *B. sonorensis* strain ACP2 and ACP production profiles. After 2.0 h of cultivation time, the lactic acid concentration in the media rose from 72 to 1338 ng mL^−1^ to acidify the medium pH, then dropped to 184 ng mL^−1^ before increasing again to its peak of 3488 ng mL^−1^ at 14 h of incubation just before the biomass peak by 2.0 h. Lactic acid production steadily declined over time until the culture period's end. However, citric and gluconic acids, which reached their maximum production levels of 1038 ng mL^−1^ at 2.0 h and 4.0 h, respectively, were consumed rapidly to achieve concentrations of 64.8 and 40 ng mL^−1^ at 10.0 h and 8.0 h, respectively. After passing through this point, the production trends of both acids remained stable with minor fluctuations until the end of cultivation. Glutamic acid, on the other hand, followed a similar trajectory to that of cell growth. Almost identically, it kept climbing till it peaked (478 ng mL^−1^) at 20 h, which is the time that *B. sonorensis* strain ACP2 growth had reached the early stationary phase, which was 2.0 h later than the peak time at which ACP production peaked, and a steady production trend was observed after this point had been passed. Based on Fig. 9c, it can be shown that the succinic and tartaric acid trends gradually grew in tandem with bacterial growth, reaching their maximum peaks (239.9 and 35.31 ng mL^−1^) at 16 h and 28 h of incubation time, respectively. The high of succinic acid coincided with the peak of bacterial biomass. At that moment, the culture medium's pH was 5.91, and the tartaric acid peak was attained when the glucose content was zero at 6.92 pH of the culture medium during the late bacterial stationary phase. As a result, pH influences bacterial proliferation in addition to the synthesis of organic acids and ACP.

Notably, after 4.0 h of cultivation, *B. sonorensis* strain ACP2 produced salicylic acid, which peaked at 2.09 ng mL^−1^ at 8.0 h of fermentation. However, due to rapid cell growth, the concentration of salicylic acid dropped to zero before rising once more at 12 h (3.888 ng mL^−1^). This finding was followed by a period of stability in the production trend up to 16 h, which matched the highest biomass point. After passing through this point, the production curve remained steady with slight oscillations until the cultivation period's end (Fig. 9c). In almost the same way, the production of malic acid was not observed for the first 6.0 h of incubation time after which it began to be produced and increased upward gradually throughout the experiment until it reached its pinnacle at 26 h, after which it became steadily stable after exceeding its peak point until the fermentation period's end.

In the precise same MRM channel, salicylic acid was found to exist; however, the hydroxybenzoic acid molecule was separated in the chromatogram due to their molecular weights being so close to each other, as displayed in Fig. 9d. The majority of organic acids were ultimately produced at *B. sonorensis* strain ACP2's stationary phase, which corresponds to the phase of ACP production. This suggests that organic acid formation is necessary for acidifying the cultural broth by lowering pH, which in turn helps to solubilize CaCO_3_, and this enhances the production of ACP. This study also showed that most of organic acid standards were identifiable in culture and that variations in their amount occurred over time.

### Thermal analysis

**Fig. 10:**
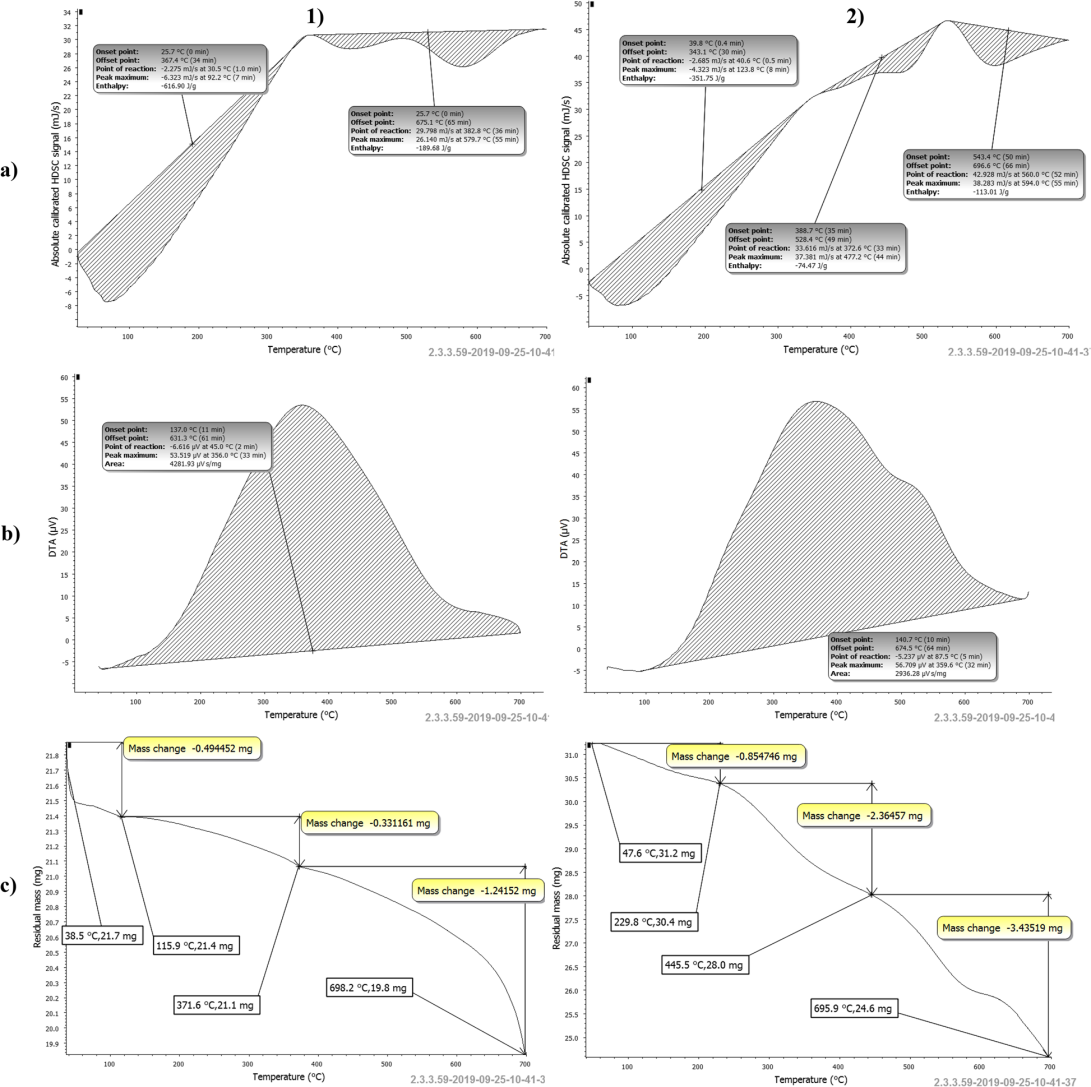
**a** DSC analysis pattern for eggshells leftover samples, 1) At zero incubation time; 2) After 32 h of incubation time; **b** DTA analysis pattern; **c** TGA analysis pattern

For thermal analysis, the residual eggshell samples were subjected to heating to 700°C in an icy nitrogen setting at 10°C min^–1^, as illustrated in Fig. 10. Two distinctive peaks could be detected on the eggshell remnant sample DSC graphs at zero incubation time. The first endothermic spike emerged at 92.0°C with a heat flux of –6.323 mJ s^–1^ and an enthalpy of –616.90 J g^–1^ due to the removal of physically adsorbed moisture. It was found that the second exothermic peak for the destruction of organic residues and lattice water, such as shell membranes and matrix protein, occurred at 579.7°C with a thermal flux of 26.140 mJ s^–1^ and entropy of –189.68 J g^–1^ as illustrated in Fig. 10a1. However, following 32 h of fermentation, a discernible change in the heat flow distribution was observed in the thermal curve, suggesting that the solubilization process had resulted in a modification of the DSC profile. With a heat flux of –4.323 mJ s^–1^ and an enthalpy value of –351.75 J g^–1^, the endothermic spike was displaced to emerge at 123.8°C, 31.8°C later than the endothermic crest that had been achieved at zero time incubation.

In terms of specific thermal capacity and flux of heat, the endothermic peak intensity was likewise less than those measured at zero time by 265.15 J g^–1^ and 2.0 mJ s^–1^, respectively. While the exothermic peak was identified at 594°C, which is 14.3°C later than those achieved at zero time, the thermal flow and enthalpy values were documented as 38.283 mJ s^–1^ and –113.01 J g^–1^, respectively, which were 12.143 mJ s^–1^ higher and 76.67 J g^–1^ lower than those observed at zero time. Figure 10a2 illustrates how these alterations in the physicochemical properties and structure of eggshell resulted from the growth of the *B. sonorensis* strain ACP2, which produced ACP, organic acids, and other metabolic byproducts and caused the disintegration of CaCO_3_ and calcium phosphate. One more exothermic peak was found at 477.2°C with a heat flow of 37.381 mJ s^–1^ and a specific heat capacity (enthalpy) of –74.47 J g^−1^ after 32 h of incubation. The breakdown of the calcium carbonate component to calcium oxide by organic and enzymatic acids, along with the release of CO_2_ as a byproduct of the disintegration process accomplished by *B. sonorensis* strain ACP2, was shown to be the source of this peak, as indicated by the following equation: $Ca{Co}_{3}\to CaO+{CO}_{2}$. The weight loss was evaluated as a function of temperature using thermogravimetric analysis. The findings obtained from DSC analysis are in reasonable agreement with those obtained by DTA analysis regarding the exothermic reaction peaks (Fig. 10b1-Fig. 10b2). As a point of comparison, the thermal decomposition of an eggshell fragment after 32 h of incubation and at zero time is demonstrated in Fig. 10c. As a result of some surface humidity and water that had been physically adsorbent in eggshell particles being lost, a slight weight loss of 2.28% with a mass change of -0.494 mg was recorded as the temperature attained 115.9°C, as shown by the three zones that characterize the thermal decomposition behaviour, as displayed in Fig. 10c1. An additional 3.65% weight loss and a mass change of -0.331 mg are seen in the second decomposition stage, which happens at temperatures between 115.9°C and 371.1°C. This decomposition step is attributable to the degradation of organic residue that is part of the eggshell waste and the outer shell membrane. Afterwards, a further 9.58% weight loss and a mass alteration of -1.241 mg are caused by the dissolution of CaCO_3_ into CaO and CO_2_ during the third stage of decomposition, which occurs between 371.1 and 698.2°C. The total weight loss of eggshells left behind after zero incubation period was 8.75% of their initial weight according to thermogravimetric analysis from 25 to 700°C. Figure 10c2, on the other hand, depicted the TGA pattern for eggshells after the fermentation process had ended at 32 h of incubation time. Weight loss was measured at 4.702%, 2.06 times more substantial than the weight loss reported at zero time as the endothermic low-amplitude crest emerged at 47.6°C and persisted until 229.8°C. This finding increased the mass change of 0.854 mg compared to the mass change witnessed at null time by 2.58 fold. In the temperature array of 229.8 to 445.5°C, an area of significant exothermic activity induced by the decomposition of organic residues was observed, leading to a second weight loss of 12.22% and a 2.364 mg mass change, which was with more by a ratio of 3.34 and 7.141, respectively, than the weight drop and shift in mass recorded at zero incubation time. In response to a rise in the dynamics of carbonate thermal breakdown, an overall weight loss of 22.88% and the ultimate mass change of 3.435 mg were noted between 445.5 and 695.9°C, which were significantly more significant than the obtained values at zero incubation time by factors of 2.388 and 1.924, respectively, as seen in Fig. 10c2. TGA analysis showed that the total weight loss of eggshells after 32 h of fermentation time was 21.15% of their initial weight, more than 2.417 times the whole weight loss seen at zero incubation time.

#### Fourier-transform infrared spectroscopy (FT-IR) and XRD analysis

**Fig. 11:**
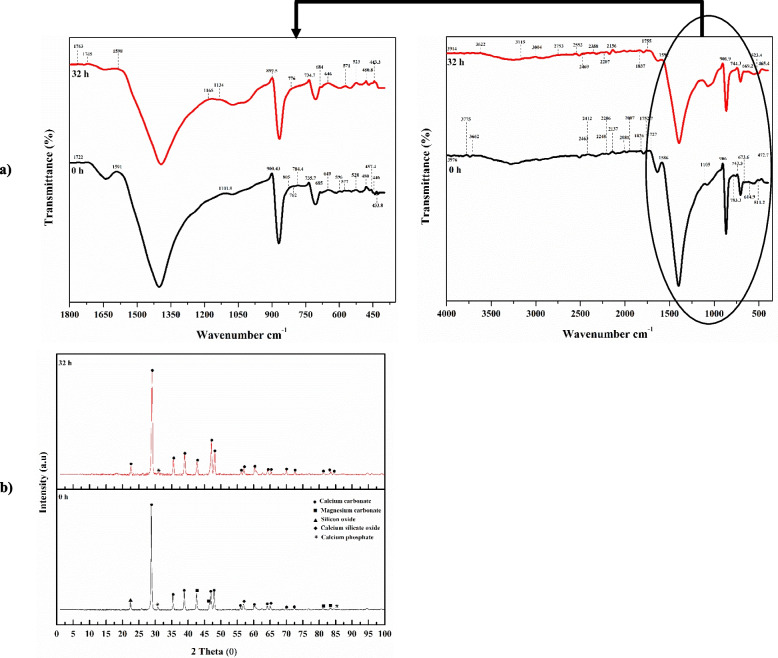
**a** FTIR pattern eggshells residual samples at 0 and 32 h incubation time; **b** XRD analysis of eggshells leftover samples at 0 h and 32 h incubation time

As seen in Fig. 11a, FT-IR analysis was performed on left eggshell samples before and throughout the incubation to detect an active group in the samples and assist in understanding the results of XRD. The spectral data indicated that the intensity of eggshell particles peaked at 1592 cm^−1^, which was highly correlated with the amount of carbonate minerals inside the eggshell matrix. Furthermore, the O–H stretching vibration of intercalated water molecules and hydrogen-bond O–H groups is accountable for the broad summit at 3622 cm^−1^; this suggests that the eggshell's hydrophilic qualities may be attributed to its surface. In addition to the O-Mg-O bending modes at 480 cm^−1^, the spectral peaks at 596 cm^−1^–577 cm^−1^ might be ascribed to the Ca-O group, according to the FTIR spectrogram of eggshell. In addition, Ca-O stretching was responsible for the 734 cm^−1^ wavenumbers. Two distinct O-Si–O peaks were seen in depth at 646 cm^−1^ and 684 cm^−1^. The absorption band at 805 cm^−1^, attributed to the asymmetric va of Si–O, was detected in an eggshell sample with zero incubation time. Moreover, silicate group vibrations may be responsible for the 784 cm^−1^ bands detected. SO_4_^−2^ is relevant for the bands seen at 685 and 1134 cm^−1^. After fermentation, the eggshell sample encompassed the vibration spectrum at 2358 cm^−1^, linked to the amino-related component's N–H stretch. Aside from that, the vibration band at 1165 cm^–1^ was the distinctive absorption peak of intercalated sulfate anions vibration, which was seen in the spectrum of the eggshell sample after the fermentation process. The occurrence of distinctive P confirmed the existence of hydroxyapatite in the eggshell samples–O stretching peaks in the wavenumber range 1101.9 cm^−1^. During fermentation, eggshell carbonate breaks down into CaO, and absorption bands of CO_3_^−2^ molecules may have shifted to higher energy levels, as seen by 899 and 528 cm^−1^ absorption bands. The carbonate group of CaCO_3_ is represented by asymmetrical C–O bending and out-of-plane and in-plane distortion modes, which may be observed at about 875 and 713 cm^−1^, respectively [25]; this observation accords with the reported carbonate data. O-Ca-O bending mode was identified by the appearance of vibrational peaks at 433 cm^−1^. On the spectral map, the spectrum bands of absorption corresponding to the Si–O-Si (*v*_*1*_) and O-C-O (*v*_*3*_) could be detected at around 776 and 2300 cm^−1^. It was determined that the O–H stretching vibrations were identified at the broad peaks of 3004 and 3115 cm^−1^ in the collected eggshell sample after the fermentation process, thereby confirming the existence of free hydroxyl groups and the bound O–H peaks of carboxylic acids. The X-ray diffraction (XRD) experiment loomed large in this discussion. The state of matter and crystalline structure of the leftover eggshells were assessed by XRD investigation both before (0 h) and following the process of fermentation (32 h). The results are displayed in the diffractogram, which is depicted in Fig. 11b. It was remarkable to note that the rhombohedron-structured calcite (CaCO_3_; PDF: 03–0569) is the primary component of finely powdered chicken eggshell waste at zero incubation time with a proportion of 63.59%. Strong and major peaks at 2Ɵ values of 28.87, 35.45, 38.96, 47.20 with the lattice plane of (024), 48.61 (116), 56.65 (211), 60.77 (214), 64.18, 69.33 with the lattice plane of (217) and 72.03 confirmed the presence of calcite (CaCO_3_). As previously stated, calcium carbonate (CaCO_3_) is visible in the eggshell sample, proving that it is the only crystalline component present in the eggshell as received. However, the major three peaks of magnesium carbonate (MgCO_3_; PDF: 02–0875) were located at 2Ɵ values of 46.93, 81.72, and 83.57 with the lattice planes of (202), (128), and (220), respectively. Moreover, calcium phosphate peaks (Ca_3_(PO_4_)_2_; PDF: 03–0691) were observed at 2Ɵ values of 30.92 and 84.11, although a single prominent peak at 2Ɵ = 22.66 in the plane of (101) suggests the existence of silicon oxide (SiO_2_; PDF: 82–1557). Eventually, two peaks supporting the presence of calcium silicate oxide (Ca_3_(SiO_4_)O; PDF: 84–0594) were seen with 2Ɵ values of 57.03 and 65.61 with the lattice planes of (203) and (004), respectively. On the other side, it was noticed that the percentage of calcite (CaCO_3_; PDF: 72–1937) dropped to 9.48% over a 32 h incubation period, highlighting the significance of organic acids and ACP in the solubilization of CaCO_3_. The major detected peak for calcium carbonate was found at a 2Ɵ value of 29.37 with the lattice plane of (104). It demonstrated that the sample's maximum value was in the (104) plane, demonstrating that the *x*- and *z*-orientation of the crystal is uniform. Furthermore, the other major peaks at 2Ɵ values of 23.03, 35.94, 39.37, 43.12, 47.45, 48.45, 56.51, 57.34, 60.61, 64.60, 65.53, 70.16, 72.81, 81.42, 83.68 and 84.71 with the lattice planes of (012), (110), (113), (202), (018), (116), (211), (122), (214), (300), (0012), (0210), (128), (2110), (134) and (226), respectively, were observed and represented the calcite (CaCO_3_) portion in eggshell waste. The occurrence of calcium phosphate (Ca_3_(PO_4_)_2_; PDF: 70–2065) was identified at a 2Ɵ value of 31.05 with the lattice plane of (217). Aside from the calcium phosphate and magnesium carbonate found in the calcified layers, eggshells include organic substances such as glycoproteins and some water trapped at the calcite crystal's grain border. Because of the processes of fermentation and dissolution by the strain under investigation, powder XRD has a detection limit of 1–2 percent for standard testing, meaning that these elements are either non-crystalline or nonexistent.

## Discussion

The current study aimed to identify a sustainable alternative for reusing and valuing eggshell waste by evaluating its ability to synthesize organic acids and ACP. According to genetic and morphological evaluation, the best-performing ACP-producing *B. sonorensis* strain ACP2 was a local bacterial strain recovered from the pulp and paper industries' wastewater. The ability of various bacteria to produce ACP has been investigated in a few studies. These bacteria include strains from the genera *Pseudomonas*, *Leclercia*, *Piriformospora*, *Serratia*, *Burkholderia*, *Pantoea*, *Citrobacter*, *Rhizobium*, and *Enterobacter* (among others) [26]. The authors of this study related the environmental feasibility of bacterial use of eggshell waste with the scaling up of ACP synthesis from *B. sonorensis* on a bench-top bioreactor scale, although, to the best of their knowledge, no prior published data had been published on any of these topics. In industrial enzyme production by microorganisms, the essential physicochemical parameters are the inoculum size, pH, and temperature. The temperature was discovered to affect extracellular enzyme production, potentially by altering the physical characteristics of the cell membrane [27]. According to the results of this investigation, the *B. sonorensis* strain ACP2 produced the highest amount of ACP (39.14 U L^−1^) when grown at 45ºC. The current study's outcomes are close to those of Behera et al. [28], who discovered that *Serratia* sp. produces the most acid phosphatase (77.87 U/ml) at 45 °C, and above the optimum temperature, the acid phosphatase production trend dropped. Nonetheless, it was found that the *B. haynesii* strain ACP1 produced the most ACP (40.8 U L^−1^) at a temperature of 50 °C. [13].

The ACP production trend steadily increased from pH 3 (36.35 U L^−1^) to pH 7 (43.11 U L^−1^) before decreasing to 35.9 U L^−1^ at pH 8.0, which was recorded in this study. The results agreed with those obtained earlier by Musarrat et al. [27], who discovered that the optimal pH range for bacteria to dissolve organic P ranged from neutral to moderately acidic. In addition, it was stated by Abdelgalil et al. [13] that an ideal ACP activity peak of 49 U L^−1^ at pH 7.0 was noted; ACP throughput fell to 47.7 U L^−1^ at pH 8.0 beyond the optimum pH. Furthermore, the original pH unadjusted media (approximately pH 7.5) produced the most ACP (56.9 U L^−1^) in comparison to the changed media with 1 N HCl (49 U L^−1^) [13]. However, a finding that contradicts the present investigation was documented by Behera et al. [28], who found that the most fabulous ACP production from *Serratia* sp. was attained at pH 5.0 (80.66 Uml^−1^) and then declined. Additionally, the findings from the current investigation indicated that the activated pre-cultures were held at 5% (v/v) inoculum size to produce the most incredible ACP output (41.8 U L^−1^). The ACP production of the *B. haynesii* strain was found to be maximum at 4.0% (v/v) but reduced as the size of the inoculum rose, according to Abdelgalil et al.'s [13] study, which made the same discovery.

A novel and inventive nutritive medium formulation was created using sequential statistical techniques to produce ACP using low-cost and easily sourced components. PBD were used to examine the effects of eleven distinct medium constituents— glucose, sodium glutamate, NH_4_NO_3_, NH_4_Cl, eggshell powder, KCl, MgSO_4_•7H_2_O, NaH_2_PO_4_, ammonium molybdate, CrCl_3_•6H_2_O, FeSO_4_•7H_2_O, corresponding to *X*_*1*_*–X*_*11*_, at the most minor and most significant factor levels on the synthesis of ACP. PBD revealed that the main variables influencing the manufacturing processes were glucose, MgSO_4_.7H_2_O, NaH_2_PO_4_, and CrCl_3_.6H_2_O. However, *B. haynesii* strain ACP1 was positively impacted by glucose, (NH4)_2_SO_4_, rock phosphate, and NaCl in the synthesis of ACP, while potassium citrate, NaNO_3_, urea, CoCl_2_•6H_2_O, CuSO_4_•5H_2_O, and NiSO_4_ showed significant adverse effects on ACP production, whereas MgCl_2_•6H_2_O had a slight influence on the output of ACP through PBD as documented by Abdelgalil et al. [13]. Moreover, Behera et al. [28] also showed that glucose and (NH_4_)_2_SO_4_ were the optimum carbon and inorganic nitrogen sources for *Serratia* sp. growth and ACP synthesis (80.66 and 80.92 U mL^−1^, respectively), out of all the carbon and nitrogen sources utilized. This finding is consistent with the present study's findings regarding the glucose item but conflicts with the existing study's conclusions regarding the (NH_4_)_2_SO_4_ item.

The most efficient combinations of the variables under study that increase *B. sonorensis* strain ACP2's synthesis of ACP were estimated via response optimization. By using the response surface approach (RSM), it is possible to refine the formulation. It will be possible to validate the projected optimal formulation using a final series of investigations. At 45°C and 200 rpm for 24 h, the ACP2 strain of *B. sonorensis* reported the maximum anticipated value of ACP (195.45 U L^−1^) in the presence of g L^−1^: NH_4_NO_3_ (0.5), glucose (25.24), eggshell powder (2), MgSO_4_•7H_2_O (0.637), NaH_2_PO_4_ (0.455), CrCl_3_•6H_2_O (0.0056) and FeSO_4_•7H_2_O (0.0015) without pH alteration. It was reported that *B. haynesii* strain ACP1 was able to achieve the highest expected value of ACP (116.5 U L^−1^) under culture conditions of 50°C and 200 rpm for 24 h in the presence of g L^−1^: glucose (23.2), (NH_4_)_2_SO_4_ (1.3117), RP (41.6), and NaCl (1.3439) without any need for a pH correction [13].

The use of sophisticated statistical optimization strategies to increase ACP output and reduce production expenses has gained popularity, and this study is the second of its sort to focus on this trend. The production efficiency of ACP was further enhanced by using scale-up bioprocessing techniques in 7 L bench-top fermenters. When *B. sonorensis* strain ACP2 growth layout was nearing the end of its stationary stage, it was found that the ACP production reached its crest (194.26 U L^−1^) with a production rate (*Q*_*p*_) of 5.023 U L^−1^ h^−1^. However, according to an earlier study by Butler et al. [29], the acid phosphatase activity produced by *Citrobacter* sp. rose around three times during exponential growth, which contradicted the present study's result that the production of ACP rose at the end of the stationary phase. Abdelgalil et al. [13] have observed that *B. haynesii* strain ACP1 produced the most considerable amount of ACP (207.6 U L^−1^) during the late exponential phase, along with the most significant ACP specific yield (222.76 U g^−1^) and production ratio *Q*_*p*_ (5.01 U L^−1^ h^−1^).

The results of this study showed that the bioreactor culture strategy produced ACP at the highest, most improved rate possible; ~ 10.0 h has reduced the ACP's time-to-production compared to its shake flask counterpart. The ACP production capacity, production rate, and specific productivity are superior to those attained by cultivating in the shake flask method. Nevertheless, a finding that contradicts the present investigation was documented by Abdelgalil et al. [13], who discovered that stirred tank bioreactors did not promote the growth of *B. haynes*ii strain ACP1 compared to shake flasks. The current investigation has shown that the *B. sonorensis* ACP2 strain could no longer produce ACP efficiently under controlled pH settings. Therefore, the most favourable setting for boosting ACP production is an unregulated pH culture state. There was an agreement between the current findings and those made previously by Abdelgalil et al. [13], who demonstrated the *B. haynesii* strain ACP1 did not thrive under controlled pH settings due to a negative impact on their growth and survival.

In the present investigation, a new formulation medium for producing ACP and organic acids was developed utilizing simple and low-cost ingredients simultaneously. *B. sonorensis* strain ACP2's ACP production was optimized sequentially, beginning with PBD (72.4 U L^−1^), progressing to OCCD (194 U L^−1^), and lastly to uncontrolled pH cultivation approach (216.37 U L^−1^). The paucity of relevant literature makes it impossible to compare our findings to those of others. *Pantoea* sp. S32 to produced ACP with a maximal activity of 69.4 U L^−1^, 3.11 times lower than the activity recorded in the current study [30]. Furthermore, *P. aeruginosa* CH01 produced the highest amount of ACP (18.4 U L^−1^) after 96 h, 11.75 times less than the strain under investigation [31].

A *Bacillus* sp. isolated from forest soil showed little activity (0.02–1.01 U) on a medium employing para-nitrophenyl phosphate as artificial organic phosphate (P_o_), according to Rahmansyah and Sudiana [32]. In Abdelgalil et al.'s [13] study, *B. haynesii* strain ACP1 produced a significant quantity of ACP despite having an activity of 207.6 U on an RP-based medium. On the other hand, strain ACP2 of *B. sonorensis* used in this investigation exhibited a notable amount of ACP, exhibiting an activity of 216.37 U in the eggshell-based medium. Meanwhile, 2.84 times less ACP activity (76 U L^−1^) was shown by *Piriformospora indica* in the RP-containing medium than in the current investigation. Furthermore, Rashad et al. [33] reported that *Azotobacter salinestris* YRNF3 produced a tiny quantity of ACP (0.054 U L^−1^) to improve the calcareous soil properties. On the other hand, *Klebsiella*, *Morganella*, and *Providencia stuartii* produced a significant amount of ACP. *Morganella morganii* and *Providencia stuartii* had 170 to 675 and 1300 to 1686 U L^−1^, respectively, which were 3.11 to 7.79 times greater than the strain under study's peak activity. The production rate for *Klebsiella* species ranged from 11.63 to 18.66 U L^−1^, which was 18.60 to 11.59 times less than the strain's maximum activity [34]. The present study revealed that under *controlled* pH conditions, ACP volumetric productivity increased gradually at a production rate (*Q*_*p*_) of 1.51, reaching a high of 64.41 U L^–1^ after 28 h. Abdelgalil et al.'s [13] earlier observations, which showed that *B. haynesii* strain ACP1's ACP volumetric productivity increased gradually over the fermentation period with a production rate (*Q*_*p*_) of 3.41, were consistent with this study's findings. At 26 h, the strain produced a high of 114.5 U L^–1^,  1.77 times higher than the activity obtained in this investigation and 2.0 h earlier than those obtained through it.

For the first time, extracellular ACP produced from *B. sonorensis* strain ACP2 using a medium made with eggshell waste is being reported using statistical optimization and scale-up techniques. In EDS, the magnitude of the X-ray emitted when an electron beam strikes an electron is utilized to determine whether elements are present. The EDS of the eggshell particles shows that the particles comprise Ca, P, Si, Al, Mg, O, S, Cl, K, and C. The results of the eggshell EDS analysis investigation by other investigators were consistent with the sample's metallic composition [35, 36]. Moreover, Hassan et al. [37] documented that the carbonized eggshell particles include C, as indicated by the EDS analysis of the ES particles, which also contain Si, O, Ca, Mg, and p.

Phosphate solubilization is a result of several microbiological processes, of which the three main ones are the phosphatase enzyme, proton extrusion, and organic acid synthesis. Phosphatases might be released from microorganisms simultaneously as organic acids to boost P solubility and mineralization by increasing hydrolytic degradation rates, according to Malboobi et al. [38]. Increasing solubilization potential may be attributed to microbial strains' simultaneous production of several organic acids. Lactic acid was discovered to be the most prevalent acid produced by the strain under study through LC–MS/MS analysis, followed by citric acid, glutamic acid, and the hydroxybenzoic acid isomer. This finding is in line with those obtained by Rashad et al. [33], who reported that *A. salinestris* YRNF3 produced lactic acid; their study offers insightful information on the mass transfer and reaction kinetics of lactic acid with calcite and dolomite, which might further knowledge of the processes behind carbonate solubilization and how they affect subsurface fluid flow and transport. Consistent with the results of the current investigation, Behera et al.'s [28] and Abdelgalil's [13] studies found that lactic acid was the most predominant acid produced during the growth of *Serratia* sp. and *B. haynesii* strain ACP1, respectively.

Moreover, among the five distinctive organic acids found in *P. ostreatus* culture broth, lactic acid was the most common, followed by citric acid, according to Maharana et al. [39]. These published results have highlighted the prevalence of lactic acid production across many microorganism species. In contrast to the current data, it was shown that among other organic acids, the solubilization of RP by *A.* *japonicas* caused the most gluconic acid synthesis [40]. The production of organic acids such as lactic, citric, and oxalic acids is the primary cause of the solubilization of CaCO_3_ and organic phosphorus in a liquid media, according to early findings [41]. Additionally, *B. liqueniformis* and *B. amyloliquefaciens* strains were demonstrated to produce mixtures of acetic, lactic, isovaleric, and isobutyric acids [13].

Organic acids and ACP activity have been shown to have a significant role in eggshell particle solubilization by the findings of morphological, functional, and thermal analysis utilizing TGA, DSC, EDS, FTIR, and XRD. When tracking fluctuations in heat flow throughout a reaction's lifespan, the differential scanning calorimetry (DSC) methodology is primarily regarded as the most efficacious technique available. Heat may move in an exothermic reaction source's direction, an endothermic reaction source's direction, or a combination of the two. Two distinct peaks were observed on the DSC charts of eggshell residual samples. The elimination of physically adsorbed moisture caused the earliest endothermic peak to arise at 92.0°C. It was found that the second exothermic peak for the destruction of organic residues and lattice water, such as shell membranes and matrix protein, occurred at 579.7°C. The findings of the DSC analysis were consistent with the observations recorded by Ferraz et al. [42]. The findings from DSC analysis are in reasonable agreement with those obtained by DTA analysis regarding the exothermic reaction peaks, according to the three zones in which the thermal decomposition behaviour is characterized. The first decomposition stage was recorded owing to the loss of some surface moisture and water that had been physically adsorbent in eggshell particles, which takes place at 115.9°C. Because of the organic residue's breakdown in the eggshell waste and the outer shell membrane, which happens at temperatures around 115.9°C to 371.1°C, there is an extra weight loss during the subsequent phase of decomposition.

Afterwards, further weight loss is caused by the dissolution of CaCO_3_ into CaO and CO_2_ during the third stage of decomposition, which occurs between 371.1 and 698.2°C. The findings are consistent with previously published data. On the other hand, Mukarami et al. [43] found that the mass loss was 2.6% up to 630°C due to the loss of volatile material. They also discovered that the CaCO_3_ breakdown accelerated with temperatures from 636 to 795°C, with Δ_m_ = 42.5%. Kristl et al. [44] gave an extremely thorough description of the decomposition process, including water loss from 25 to 226 °C (Δ_m_ = 1.03%), organic matter decomposition from 226 to 574 °C (Δ_m_ = 3.77%), and calcium carbonate (CaCO_3_) breakdown between 574 and 769 °C (Δ_m_ = 42.17%). Furthermore, Naemchan et al. [45] reported a comparable mass loss trend between 100 and 600 °C, which they associated with the decomposition of organic protein components and water loss, followed by substantial mass loss in the 600–850 °C range.

The magnitude and location of spectral bands, as well as the presence and absence of certain peaks, were significantly influenced by the fermentation and dissolution processes, according to an FTIR investigation. Following the solubilization process, the band locations and intensity levels significantly changed. The O–H stretching vibration of intercalated water molecules and hydrogen-bond O–H groups is responsible for the peak at 3622 cm^−1^, which was noticed. This finding aligns with data that has been previously published. The OAH vibrating stretching and twisting hydroxyl groups observed in Ca(OH)_2_ were identified as the cause of the peak of absorption that appeared at 3642 cm^−1^ for calcined eggshells [46, 47]. Furthermore, according to a study by Abdelgalil et al. [13], bending phases of vibrational of H–OH were observed at 3611 and 1628 cm^−1^, where water-adsorbed peak intensities were identified.

The FTIR spectrogram of eggshells of the present study suggests that the Ca-O group may be accountable for the spectrum crests at 596 cm^−1^–577 cm^−1^ in addition to the O-Mg-O twisting modes at 480 cm^−1^. Additionally, the 734 cm^−1^ wavenumbers were caused by Ca-O stretching. Akermanite rocks heated at 1200 ◦C showed O-Ca-O twisting phases at 415 cm^−1^ and O-Mg-O twisting forms at 486 cm^−1^, according to Choudhary et al. [48]. The Ca-O group is present in the peak at 586 cm^−1^, while the O-Si–O peaks were found at 640 and 682 cm^−1^. The 852, 935, and 974 cm^−1^ summits display the Si–O expanding phases.

Lines at 966, 1045, and 1097 cm^−1^ are developed by asymmetric and antisymmetric expansions of (PO_4_)^−3^ groups, while bands at 474, 578, and 605 cm^−1^ are brought about via symmetric and antisymmetric distortions of (PO4)^−3^ categories, according to Bachouâ et al. [49]. According to Abdelgalil et al. [13], the anti-symmetrical resonance of the (CO_3_)^−2^ set is responsible for the spikes noticed at 870 and 1420–1426 cm^−1^ in both the calcite and dolomite phases, respectively. Moreover, according to Abdelgalil et al., silicate group vibrations may be the root of the bands observed in the 788 cm^−1^ regions. Furthermore, bands appearing at 672 cm^−1^ are associated with SO_4_^–2^, while the peaks at 1642 cm^−1^ may be related to the twisting and antisymmetric vibrations of stretching of OH^–^ [13]. The present finding reveals that the absorption band at 805 cm^−1^, attributed to the asymmetric va of Si–O, was detected in a sample with zero incubation time eggshells. Moreover, silicate group vibrations may be responsible for the 784 cm^−1^ bands seen. On the other hand, Abdelgalil et al. [13] documented that the solubilization process for rock phosphate by *B. haynesii*, which helps to leach Si from the rock matrix, prompted the 518 cm^−1^ absorption peak, whereby the RP leftovers' Si–O–Si asymmetric bending matched. The O–C–O (*v*_*3*_) and Si–O–Si (*v*_*1*_) absorption spectra were seen at 2400 cm^−1^ and 539–818 cm^−1^, respectively, after sample solubilization. X-ray diffraction (XRD) was employed to analyze residual samples of finely crushed eggshell powder, and the crystalline direction and functioning features of the organic matrix elements in eggshells were assessed. The XRD pattern in the present investigation was in line with XRD patterns obtained by Ummartyotin et al. [50] and Laohavisuti et al. [4], confirming the presence of calcite in the eggshell. Minakshi et al. [36] reported that peaks that could be indexed to CaCO_3_ were found by XRD analysis, indicating that the crushed powder did not include any further contaminants. The sample's distinctive calcium carbonate peaks, which agree with the published pattern, demonstrate that CaCO_3_ is the only crystalline phase in the eggshells as they were received. Moreover, Laohavisuti et al. [4] reported that diffraction lines in as-prepared tricalcium phosphate primarily correspond to beta-tricalcium phosphate (Ca_3_(PO_4_)_2_, PDF no. 70–2065), which is in line with the current finding. However, according to Awogbemi et al. [51], the XRD patterns of market CaO, impregnated CaO, and CaO from discarded chicken eggshells all had spikes at 2θ values of 35, proving the presence of CaO.

## Conclusions

To deal with today's increasing volume of eggshell waste, agriculture, food businesses, and communities face a significant challenge. A new method of recycling trash was proposed in this study to alleviate the eggshell issue faced by businesses and communities. The current study explored the possibility of using eggshell waste to produce organic acids and ACP to devise an environmentally friendly approach to recycling and economically utilize it. The genetic and morphological analysis helped identify the most effective ACP-producing *B. sonorensis strain*, ACP2, as a native bacterial strain recovered from the pulp and paper industries' sewage. Sequential statistical methodologies were used to design a novel and unique nutritious medium composition that included affordable and easily accessible components needed to produce ACP. Through PBD, it was determined that the most substantial variables influencing the procedures for production were glucose, MgSO_4_.7H_2_O, NaH_2_PO_4_, and CrCl_3_.6H_2_O. The efficiency of producing ACP was further enhanced by using scale-up bioprocessing techniques in a 7 L bench-top fermentor. The *B. sonorensis* ACP2 strain lost its capacity to produce ACP effectively under controlled pH cultivation conditions. According to the present study's findings, an uncontrolled pH cultivation condition is the best option to optimize ACP production. Starting with PBD (72.4 U L^−1^), the ACP production by *B. sonorensis* strain ACP2 was improved successively, moving on to OCCD (194 U L^−1^) and then to unmanaged pH cultivation (216.37 U L^−1^). Lactic acid was discovered to be the most prevalent acid produced by the strain under study through LC–MS/MS analysis, followed by citric acid, glutamic acid, and the hydroxybenzoic acid isomer. Organic acids and ACP activity have been shown to have a significant role in eggshell particle solubilization by the morphological and functional analysis findings utilizing TGA, DSC, SEM, EDS, EDS, FTIR, and XRD.

### Supplementary Information

**Supplementary Material 1.**

## Data Availability

All data produced during this study are included in this published article.

## Abbreviations

ACP: Acid Phosphatase
AAS: Atomic Absorption Spectrometry
ANOVA: Analysis of Variance
AT%: Atomic Percentage
B: Bacillus
CCD: Central Composite Design
CV: Coefficient of Variation
DF: Desirability Function
DSC: Differential Scan Calorimeter
EDS: Energy-Dispersive Spectroscopy
FTIR: Fourier-Transform Infrared Spectroscopy
JCPDS: Joint Committee on Powder Diffraction Standards
h: Hour
LB: Luria–Bertani
NPP: *ρ*-Nitrophenyl phosphate
PBD: Plackett–Burman Design
PDP: Phenolphthalein diphosphate tetrasodium salt
PKV: Pikovskaya
PRESS: Predicted Residual Sum of Squares
TGA: Thermogravimetric Analyzer
RP: Rock Phosphate
RSM: Response Surface Methodology
OCCD: Orthogonal Central Composite Design
STRs: Stirred-Tank Reactors
XRD: X-Ray Diffraction
