# Supplementary material for: Eggshell waste bioprocessing for sustainable acid phosphatase production and minimizing environmental hazards
Source: J Biol Eng. 2024 Apr 8;18:26. doi: 10.1186/s13036-024-00421-8 (PMC11003023; doi:10.1186/s13036-024-00421-8)
Supplement: Supplementary file 1 — Supplementary Material 1. [file 13036_2024_421_MOESM1_ESM.docx]

**Supplementary Data**

**Journal of Biological Engineering**

**Eggshell waste bioprocessing for sustainable acid phosphatase production and minimizing environmental hazards**

**Soad Abubakr Abdelgalil^1*^^[[1]](#footnote-1)^, Mohamed Mohamed Yousri Kaddah^2^, and Gaber Attia Abo-Zaid^1^,**

^1^ Bioprocess Development Department, Genetic Engineering and Biotechnology Research Institute (GEBRI), City for Scientific Research and Technological Applications, Alexandria, Egypt

^2^ Pharmaceutical and Fermentation Industries Development Center, City for Scientific Research and Technological Applications, Alexandria, Egypt

**Table S1.** Linear regression equations and analytical parameters of the organic acids by the proposed LC-MS/MS method

| Compound | Linearity range ng/mL | Regression equation | Correlation coefficient (*r*) | LOD | LOQ |
| --- | --- | --- | --- | --- | --- |
| Lactic acid | 10 – 1600 | *y* = (119.1 *x* + 1.75) × 10^5^ | 0.9991 | 1.89 | 6.29 |
| Pyruvic acid | 25 – 2500 | *y* = (71.9 *x* + 2.16) × 10^4^ | 0.9990 | 4.70 | 15.67 |
| Gluconic acid | 1 – 75 | *y* = (57.2 *x* + 7.11) × 10^3^ | 0.9995 | 0.03 | 0.10 |
| Malic acid | 0.2 – 80 | *y* = (23.2 *x* + 1.43) × 10^4^ | 0.9982 | 0.04 | 0.13 |
| Tartaric acid | 1 – 500 | *y* = (4.33 *x* + 2.63) × 10^4^ | 0.9992 | 0.05 | 0.16 |
| Citric acid | 0.3 – 300 | *y* = (9.38 *x* + 3.72) × 10^4^ | 0.9991 | 0.15 | 0.51 |
| Salicylic acid | 0.2 – 20 | *y* = (39.26 *x* + 3.82) × 10^4^ | 0.9991 | 0.03 | 0.09 |
| Succinic acid | 0.3 – 30 | *y* = (1.81 *x* + 1.31) × 10^5^ | 0.9979 | 0.01 | 0.03 |
| Glutamic acid | 0.6 – 60 | *y* = (4.46 *x* + 1.64) × 10^4^ | 0.9992 | 0.03 | 0.12 |

**Table S2.**  Intraday accuracy and precision of the organic acids by the proposed LC-MS/MS method

| Organic acid | Concentration level | Intra-day accuracy and precision | | | |
| --- | --- | --- | --- | --- | --- |
|  |  | Amount taken (ng/mL) | Found concentration (mean ± SD) | Accuracy (%) | CV (%) |
| Lactic acid | LQC | 20 | 19.92 ± 0.42 | 99.58 | 2.09 |
|  | MQC | 800 | 798.95 ± 23.55 | 99.87 | 2.95 |
|  | HQC | 1200 | 1235.5 ± 13.44 | 102.96 | 1.09 |
| Pyruvic acid | LQC | 100 | 107.55 ± 2.62 | 107.55 | 2.43 |
|  | MQC | 1250 | 1323 ± 1.41 | 105.84 | 0.11 |
|  | HQC | 2000 | 1974.5 ± 10.61 | 98.73 | 0.54 |
| Gluconic acid | LQC | 3 | 2.86 ± 0.08 | 95.23 | 2.67 |
|  | MQC | 40 | 39.55 ± 0.60 | 98.86 | 1.52 |
|  | HQC | 60 | 59.98 ± 2.00 | 99.96 | 3.33 |
| Malic acid | LQC | 0.8 | 0.77 ± 0.05 | 95.95 | 6.65 |
|  | MQC | 40 | 41.45 ± 0.63 | 103.61 | 1.52 |
|  | HQC | 70 | 70.90 ± 0.85 | 101.29 | 1.20 |
| Tartaric acid | LQC | 2 | 2.06 ± 0.06 | 102.88 | 3.06 |
|  | MQC | 250 | 249 ± 2.83 | 99.60 | 1.14 |
|  | HQC | 400 | 395.55 ± 14.64 | 98.89 | 3.70 |
| Citric acid | LQC | 1 | 0.99 ± 0.07 | 99.09 | 6.86 |
|  | MQC | 150 | 146.75 ± 4.03 | 97.83 | 2.75 |
|  | HQC | 250 | 248.75 ± 8.98 | 99.5 | 3.61 |
| Salicylic acid | LQC | 0.8 | 0.85 ± 0.004 | 106.57 | 0.44 |
|  | MQC | 10 | 10.15 ± 0.27 | 101.49 | 2.66 |
|  | HQC | 16 | 16.6 ± 0.08 | 103.75 | 0.51 |
| Succinic acid | LQC | 0.6 | 0.67 ± 0.02 | 111.72 | 2.68 |
|  | MQC | 15 | 14.94 ± 0.45 | 99.57 | 2.98 |
|  | HQC | 25 | 24.70 ± 0.85 | 98.8 | 3.44 |
| Glutamic acid | LQC | 2.4 | 2.64 ± 0.13 | 110 | 4.82 |
|  | MQC | 30 | 31.26 ± 1.42 | 104.18 | 4.55 |
|  | HQC | 48 | 47.85 ± 1.94 | 99.68 | 4.06 |

**Table S3.** Mass spectrometry parameters for the optimized MRM transitions of the selected organic acids

| Compound | Q1 Mass (Da) | Q3 Mass (Da) | Dwell time (msec) | DP (V) | CE (V) | CXP (V) |
| --- | --- | --- | --- | --- | --- | --- |
| Lactic acid | 88.622 | 43.000 | 175.00 | -105.00 | -16.00 | -7.00 |
| Pyruvic acid | 86.943 | 43.000 | 100.00 | -50.00 | -12.00 | -11.00 |
| Gluconic acid | 194.958 | 129.100 | 100.00 | -70.00 | -18.00 | -9.00 |
| Maleic acid | 114.917 | 71.000 | 150.00 | -60.00 | -14.00 | -9.00 |
| Tartaric acid | 148.697 | 87.000 | 100.00 | -60.00 | -18.00 | -9.00 |
| Citric acid | 190.922 | 111.000 | 100.00 | -60.00 | -18.00 | -9.00 |
| Salicylic acid | 136.944 | 93.000 | 150.00 | -120.00 | -24.00 | -9.00 |
| Oxalic acid | 88.947 | 61.000 | 100.00 | -70.00 | -12.00 | -9.00 |
| Succinic acid | 116.888 | 72.900 | 100.00 | -50.00 | -18.00 | -9.00 |
| Glutamic acid | 145.966 | 128.000 | 100.00 | -75.00 | -14.00 | -11.00 |

**Table S4.** Mass spectrometer main working parameters of ion source for the selected organic acids

| Parameter | Value |
| --- | --- |
| Curtain gas (psi) | 20 |
| Ion-spray voltage (V) | -3000 |
| Temperature C° | 250 |
| Ion source gas 1 (psi) | 45 |
| Ion source gas 2 (psi) | 45 |
| Entrance potential (V) | -10 |
| Mode of analysis | Negative |

**Table S5.** Gradient elution program for determination of organic acids

| No | Time (min) | Flow (mL/min) | Frac A (%) | Frac B (%) |
| --- | --- | --- | --- | --- |
| 1 | 0 | 0.35 | 70 | 30 |
| 2 | 1.5 | 0.35 | 70 | 30 |
| 3 | 8 | 0.35 | 0 | 100 |
| 4 | 13 | 0.35 | 0 | 100 |
| 5 | 13.5 | 0.35 | 70 | 30 |
| 6 | 15 | 0.35 | 70 | 30 |

**Figure S1**. ESI-MS/MS spectra of the molecular ions of lactic acid under the negative mode

**Figure S2**. ESI-MS/MS spectra of the molecular ions of pyruvic acid under the negative mode

**Figure S3**. ESI-MS/MS spectra of the molecular ions of Gluconic acid under the negative mode

**Figure S4**. ESI-MS/MS spectra of the molecular ions of maleic acid under the negative mode

**Figure S5**. ESI-MS/MS spectra of the molecular ions of tartaric acid under the negative mode

**Figure S6.** ESI-MS/MS spectra of the molecular ions of citric acid under the negative mode

**Figure S7.** ESI-MS/MS spectra of the molecular ions of salicylic acid under the negative mode

**Figure S8.** ESI-MS/MS spectra of the molecular ions of oxalic acid under the negative mode

**Figure S9**. ESI-MS/MS spectra of the molecular ions of succinic acid under the negative mode

**Figure S10**. ESI-MS/MS spectra of the molecular ions of glutamic acid under the negative mode

1. Correspondence and requests for materials should be addressed to:

   Soad A. Abdelgalil, New Borg El-Arab City, Universities and Research Institutes Zone, Post 21934, Alexandria, Egypt

   Tel: 00203 4593420, Fax: 00203 4593423, mobile: (+20)1009795592

   Email: [Soad_abu_bakr@yahoo.com](mailto:Soad_abu_bakr@yahoo.com)

   ORCID: 0000-0001-7902-3392 [↑](#footnote-ref-1)
